# Evidence-Based Process for Prioritizing Positive Behaviors for Promotion: Zika Prevention in Latin America and the Caribbean and Applicability to Future Health Emergency Responses

**DOI:** 10.9745/GHSP-D-19-00188

**Published:** 2019-09-23

**Authors:** Jessie Pinchoff, Arianna Serino, Alice Payne Merritt, Gabrielle Hunter, Martha Silva, Priya Parikh, Paul C. Hewett

**Affiliations:** a Population Council, New York, NY, USA.; b United States Agency for International Development, Washington DC, USA.; c Johns Hopkins Center for Communication Programs, Baltimore MD, USA.; d Tulane University, New Orleans, LA, USA.

## Abstract

Since the 2015 Zika outbreak in Latin America and the Caribbean, a plethora of behavior change messages have been promoted to reduce Zika transmission. One year after the United States Agency for International Development (USAID) initiated its Zika response, more than 30 variants of preventive behaviors were being promoted. This situation challenged social and behavior change (SBC) programming efforts that require a coordinated response and agreed upon set of focus behaviors to be effective. To support USAID implementing partners in harmonizing prevention efforts to reduce Zika infection, we developed an evidence-based process to identify behaviors with the highest potential to reduce Zika infection and transmission. We compiled a full list of behaviors and selected the most promising for a full evidence review. The review included systematic keyword searches on Google Scholar, extraction of all relevant published articles on *Aedes*-borne diseases between 2012 and 2018, review of seminal papers, and review of gray literature. We examined articles to determine each behavior's potential effectiveness in preventing Zika transmission or reducing the *Aedes aegypti* population. We also developed assessment criteria to delineate the ease with which the target population could adopt each behavior, including: (1) required frequency; (2) feasibility of the behavior; and (3) accessibility and cost of the necessary materials in the setting. These behaviors were refined through a consensus-building process with USAID's Zika implementing partners, considering contextual factors. The resulting 7 evidence-based preventive behaviors have high potential to strengthen SBC programming's impact in USAID's Zika response: (1) apply mosquito repellent, (2) use condoms during pregnancy, (3) remove standing water, (4) cover water storage containers, (5) clean/remove mosquito eggs from water containers, (6) seek antenatal care, and (7) seek family planning counseling. This case study documents a flexible process that can be adapted to inform the prioritization of behaviors when there is limited evidence available, as during many emergency responses.

## Abstract (precis)

To maximize the impact of Zika prevention programming efforts, a prioritization process for social and behavior change programming was developed based on a combination of research evidence and programmatic experience. Prioritized behaviors were: application of mosquito repellent, use of condoms, removing unintentional standing water, covering and scrubbing walls of water storage containers, seeking prenatal care, and seeking counseling on family planning if not planning to get pregnant.

Résumé en español al final del artículo.

## Introduction

Zika virus is a communicable disease primarily transmitted by the *Aedes aegypti* mosquito, a vector that also transmits other arboviruses including dengue, chikungunya, West Nile virus, and yellow fever. The first outbreak of Zika detected in the Americas occurred in 2015, with a spike in suspected congenital malformations and other neurological complications such as Guillain-Barré syndrome.^1^ By August 3, 2017, there were approximately 217,000 confirmed Zika cases, and about 3,400 cases of associated congenital Zika syndrome.^2^ Zika is now considered endemic throughout Latin America and the Caribbean (LAC), parts of Africa, and Asia. Between 29% and 82% of Zika infections are asymptomatic according to the U.S. Centers for Disease Control and Prevention (CDC).^3^ Infection during pregnancy is linked to congenital Zika syndrome in newborns, which is characterized by severe microcephaly (small head size), decreased brain tissue mass, and subcortical calcification.^4^ Other health abnormalities, including developmental delays, associated with the Zika virus have been reported. Research on the impact of the virus on mothers and children is ongoing.^4^

The outbreak in LAC demanded a concerted regional response, given the wide distribution of the mosquito vector, the lack of population-level immunity, the absence of a vaccine or rapid diagnostic test, uneven access to water due to low quality water and sanitation infrastructure, water shortages, lack of information about the disease, and inadequate health systems to respond to the health impacts.^1^ On February 1, 2016, the World Health Organization declared Zika a Public Health Emergency of International Concern.^5^ The United States Agency for International Development (USAID) and other U.S. government entities and international partners began working together through existing country systems to reduce the risk of new Zika infections, particularly in pregnant women, and to provide care for those affected through interventions in vector control, social and behavior change (SBC), and health service delivery.^6^ The focus for this process was SBC for individuals, households, and communities in Zika-affected regions, only.

USAID's SBC programming was comprised mainly of mass and social media, community engagement, and interpersonal communication, with the goal to “raise awareness, reduce misinformation, and address the barriers that prevent individuals, families, and communities from practicing lifesaving behaviors to improve health outcomes.”^7^ The SBC literature suggests that behavior change is more likely to occur when clear and concise messaging is repeated frequently through multiple channels.^8^^–^^9^ When too many preventive behaviors are promoted or messages lack precision, adopting prevention behaviors can be inhibited or done in a way that is either ineffective or counterproductive.^8^ Messaging can be particularly challenging during emergency responses when data may be unavailable to inform programming and time constraints inhibit collective planning, leading to the promotion of messages before a concerted and harmonized response can be organized. In a non-systematic, rapid desk review of SBC messages approximately 1 year after the USAID Zika response began, we identified more than 30 variants of prevention behaviors that were being promoted. The prevention messages for these behaviors were not consistently presented, lacked cohesion in their packaging, and offered little specificity regarding how the behavior should be implemented to effectively reduce Zika infection and transmission. Too many behaviors with insufficiently specific instructions in the messages could have resulted in confusion, information overload, and incorrect performance of the behaviors among individuals and communities. The behaviors promoted were also not always based on available evidence around their effectiveness in relation to Zika transmission.

Facing an outbreak of a disease new to the Americas, public health institutions and organizations found themselves conducting research while simultaneously launching interventions and programs. At the time programs were rolling out, there were limited data to guide SBC programming and messaging for the most effective preventive actions for individuals and communities. These circumstances often led to a lack of cohesion in promoted behaviors and SBC messages. To more effectively coordinate the Zika response among implementing partners and increase the rate of behavior adoption among target populations, the Breakthrough ACTION + Research Projects, in collaboration with USAID, developed an evidence-based process to identify priority behaviors with the highest potential for preventing Zika acquisition and transmission. Stakeholders across disciplines and involved in various levels of programming were engaged throughout to ensure buy-in, harmonize priority behaviors and their SBC messages, and ensure a more effective Zika response. Existing research could be leveraged because the transmission dynamics for Zika were similar to other arboviruses and sexually transmitted infections; preventive behaviors targeting the vector (the *Aedes aegypti* mosquito) and practices to reduce sexual transmission had already been identified in the literature and could be assessed for Zika. Understanding the transmission dynamics was critical to identifying behaviors to consider for prevention. The exercise, referred to as the “behavior prioritization process,” focused on a range of individual- and household-level behaviors to reduce the risk of Zika acquisition and transmission. Messages were developed by partners based on the set of behaviors identified and prioritized in this process.

An evidence-based process identified priority behaviors with the highest potential for preventing Zika.

Establishing an evidence base and a refined set of preventive behaviors tailored to the specific context can greatly improve the success of SBC programming by reinforcing promotion of consistent behaviors and using evidence to add specificity to the desired actions. The process combined available evidence and a consensus-building approach to allow for adaptation based on local context. This article summarizes our experience in prioritizing the behaviors with highest potential for Zika prevention, identifies specific target audiences for each behavior, and documents the design and implementation of the behavior prioritization process developed to achieve these aims in a flexible way. We also consider the applications of this process for strengthening future public health emergency responses.

## Methods

A list of more than 30 Zika preventive behaviors (or their variants) that USAID implementing partners across USAID-supported countries were promoting was compiled by informally reviewing numerous project materials and documents. All of these preventive behaviors were related to the transmission dynamics of Zika virus—transmission by a vector (*Aedes aegypti*) that also transmits arboviruses such as dengue and sexual transmission. A team of experts in SBC programming and vector control was enlisted to categorize and refine the behaviors. All of the Zika prevention messages were first grouped together by behavior to create a condensed version of about 15 behaviors. Through an iterative review process including experts and discussions with partners, the list was distilled to 7 key behaviors (Table 1). Behaviors were excluded if they had limited effectiveness preventing *Aedes aegypti*-borne diseases (such as Zika) or reducing *Aedes aegypti* mosquito populations (after a quick literature scan and input from experts) or due to other criteria.

**TABLE 1.** The 7 Zika Preventive Behaviors Selected for Prioritization

Behavior	Summary of Evidence
*Personal Protection*	
Applying mosquito repellent (DEET, picaridin, IR3535, or lemon eucalyptus oil, only), using each product as directed, for duration of pregnancy, to reduce risk of Zika transmission through mosquito bites.	Application of mosquito repellent is highly efficacious in preventing mosquito bites, and thus the potential of vector transmission of Zika to an individual. This behavior is within the control of pregnant women and their male partners. Users should be thoroughly counseled on proper product application. Women intending to become pregnant should also consider using repellent.
Using condoms to prevent sexual transmission of Zika in pregnancy.	Condom use to prevent sexual transmission of Zika is highly efficacious, but sexual transmission may be a small portion of overall transmission. This behavior should be prioritized for pregnant women and their partners because pregnant women are at risk for negative pregnancy outcomes.
*Household and Community Vector Control*	
Regularly removing unintentional standing water both inside and outside the house and in communal areas.	This is a potentially efficacious behavior to reduce mosquito populations, and thus reduce the potential for individual- and population-level risk of Zika transmission. Promotion of the behavior must be accompanied by specific, focused instructions that target the highest density breeding sites and be conducted weekly in homes and communal areas to be effective. Efficacy is highest in areas where there is strong community engagement, including active mosquito searches in homes and communities and awareness of the mosquito life cycle.
Covering water storage containers at all times with a tight-fitting cover that does not warp or touch the water.	Covering long-term water storage containers has moderate potential efficacy in reducing breeding sites if a tight- fitting, long-lasting lid is available. Covering short-term water storage containers has less potential efficacy, as frequent lid use can result in wear and tear and render the lids ineffective or counterproductive.
Scrubbing walls of water storage containers weekly to remove mosquito eggs.	Scrubbing walls of water storage containers weekly is efficacious in removing mosquito eggs and can thus reduce the potential for individual- and population-level risk of Zika transmission. However, the specific cleaning steps that eliminate mosquito eggs must be explicitly described.
*Behaviors That Enable Prevention*	
Seeking antenatal care to monitor pregnancy and discuss Zika risk and prevention.	Seeking antenatal care enables providers to counsel pregnant women on Zika prevention, which can increase the chances of pregnant women taking protective measures and reducing the risk of vertical transmission of Zika from mother to child.
Seeking counseling from a trained provider on modern family planning methods if not planning on getting pregnant.	Family planning use (for those not intending on getting pregnant) is directly linked to reducing the risk of vertical transmission of Zika. Family planning counseling should be done by a trained health care provider.

In determining the most promising behaviors to review further, behaviors were excluded from the list if:

1. The behavior was largely outside the control of the individual or household (e.g., indoor residual spraying or applying larvicide, which require trained technicians).
2. There was limited evidence of the behavior's efficacy (e.g., bed net use as the *Aedes* mosquito mainly bites in the daytime).
3. The behavior had only been implemented in a geographically limited pilot stage intervention (e.g., larvivorous fish in water storage containers).
4. The behavior was not supported by USAID (i.e., USAID was not procuring or distributing required materials to carry out the behavior) because of the lack of effectiveness of the behavior (e.g., bed nets are not considered effective for Zika because of the vector behavior) or because it was not feasible within the scope of the program (e.g., installing screens on windows).

Table 2 lists the excluded behaviors and reasons for not including them.

**TABLE 2:** Zika Preventive Behaviors Not Selected for Full Evidence Review and Reasons for Exclusion

Behavior	Outside Locus of Control	Limited or No Evidence of Effectiveness	Challenging in This Setting	Behavior Is in Pilot Phase	USAID Not Supporting	Summary
Use of insecticide-treated bed nets		✓			✓	This behavior has limited efficacy, as most people sleep during the night and *Aedes aegypti* mosquitoes bite mainly during the day, limiting the time nets might provide Zika protection to daytime naps. Additionally, USAID is not procuring mosquito nets for Zika since they are not effective because of the daytime biting behavior of *Aedes* mosquitoes.
Wearing long sleeves, light colors		✓	✓		✓	In the climate where Zika is transmitted, implementing this behavior with sufficient consistency (all day, every day) is unlikely to be feasible, reducing its potential to make an important contribution to Zika prevention. There is also limited evidence that wearing regular clothing that has not been treated with insecticide is effective in preventing mosquito bites.
Application of larvicide	✓			✓		While considered highly efficacious, larvicides should be applied by vector control technicians, rather than household members, so control over implementation of this behavior does not lie at the household level.
Larvivorous fish	✓			✓	✓	Application of larvivorous fish to water storage containers is still in the pilot phase; limited data available on efficacy. Additionally, USAID is not procuring larvivorous fish, and the behavior is outside the locus of household control since it is currently being done by vector control specialists who visit the home.
Indoor residual spraying	✓	✓		✓		This behavior is implemented by vector control technicians and therefore does not lie within the control of the household. There is limited literature on the efficacy of this intervention as it is traditionally only used for *anopheline* mosquitoes; some pilots are in progress to test for effectiveness for *Aedes* mosquitoes.
Use of insecticide-treated curtains/screens					✓	There is some evidence that insecticide-treated curtains or screens are effective in preventing *Aedes* abundance indoors; however, USAID is not procuring these.
Use of coils to repel mosquitoes		✓			✓	Efficacy appears limited upon initial review, with some studies even suggesting they increase dengue risk.
Planting basil plants		✓				While some research suggests that essential oils extracted from plants may have a repellent effect, no studies were identified that assess the repellent effect of basil plants.

At this stage, 5 behaviors were selected for a full evidence review that fell into the broad categories of (1) personal protection and (2) household and community vector control. A third category, prevention-enabling behaviors, was created for 2 additional behaviors that did not undergo a full evidence review here but were still recommended. Prevention-enabling behaviors create the potential for exposure to Zika prevention counseling by trained and trusted health care providers and include antenatal care and family planning counseling. Investigation of enabling behaviors was not pursued in the next phase of the process, as extensive existing literature supported the efficacy of antenatal care and family planning counseling on uptake of sexual and reproductive health services (generally), and their promotion was already ongoing through reproductive health programs in the region. Although it is not necessarily clear how the Zika-specific context may have affected uptake of these behaviors, there was no research available at the time to review.

### Evidence Review

To conduct a systematic evidence review for the 5 personal protection and vector control preventive behaviors, Google Scholar results were compiled for articles on *Aedes*-borne diseases published between 2012 and 2018. Any seminal papers published before 2012 and Zika-related gray literature and unpublished data (from sources including UNICEF, the CDC, and USAID implementing partners) were also reviewed. Because of the recency of the Zika outbreak, a limited number of relevant papers had reached publication, so relevant articles on any *Aedes*-borne diseases (dengue, chikungunya, yellow fever, West Nile) were also compiled. Literature on malaria was excluded because it is transmitted by *Anopheles* mosquitoes, not *Aedes*, calling for different interventions. A PRISMA diagram (Figure) shows the selection criteria and screening process results for articles included in the review. Each article was summarized in an annotated bibliography.

**FIGURE:**
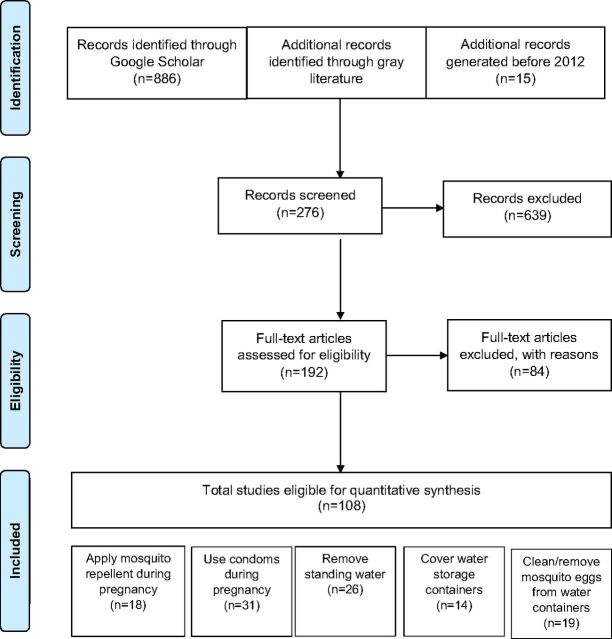
PRISMA Diagram of Process for Considering Eligibility in the Evidence Review

As noted in the top panel of Table 3, each behavior's efficacy and, if available, effectiveness in preventing Zika transmission were investigated through the literature review process. We considered a behavior efficacious if it had one of the following impacts: a reduction in mosquito bites, a reduction in the mosquito population (as measured by number of eggs, pupae, or adult mosquitoes), or a reduction in the sexual transmission of Zika. We considered a behavior effective if programs promoting the behavior had an impact on the outcome at the population level and/or measured a public health impact. The effectiveness or public health impact was not always measured or reported in the published literature; in those cases, other sources were explored for reasonably extrapolating this information. If gray literature was available, this was explored. Otherwise, logical assumptions were tentatively made; for example, if a study found that mosquito abundance was reduced, we extrapolated that Zika transmission may also be reduced.

**TABLE 3.**
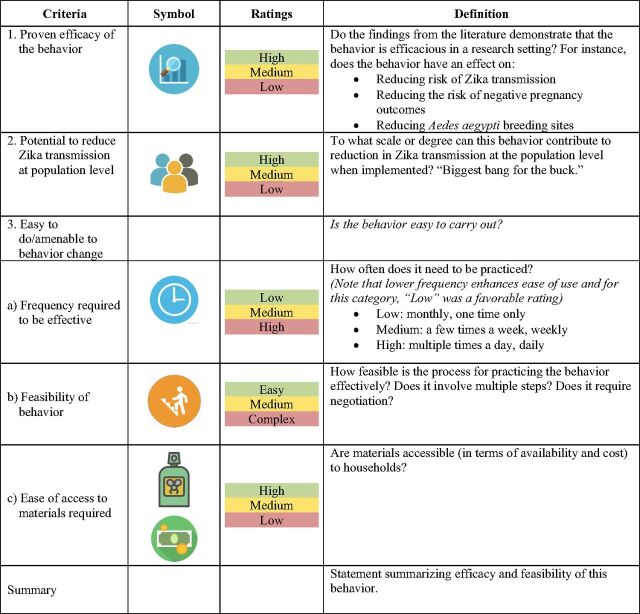
Criteria Considered for Each Priority Zika Preventive Behavior to Gauge Its Efficacy, Effectiveness, and Multiple Aspects of Feasibility

The evidence regarding efficacy and effectiveness from the literature for each behavior was assessed against each criterion as being “high,” “medium,” or “low.” The evidence was also qualitatively weighted by the rigor of the studies reviewed and how recently the study was conducted. Our literature review also considered the locus of control (who was primarily responsible for implementing the behavior); how the outcome was measured (e.g., number of mosquito bites, population density); whether programs targeted specific sub-populations (e.g., pregnant women, male partners); and whether interventions targeted multiple behaviors (e.g., larvicide application, removing stagnant water). These additional factors were considered to guide interpretation of the study findings based on context and better understand the generalizability of results (e.g., if a study assessed multiple behaviors being promoted at once and the impact of a single behavior could not be isolated). If the article had insufficient detail, we also contacted research authors to clarify the specific steps required in the behaviors assessed in their studies.

### Programmatic Assessment

In addition to efficacy and program effectiveness criteria, a third criterion was developed to assess whether the behavior was easy to do and amenable to change to consider the contextual realities of how behaviors were being promoted and adopted. As noted in the bottom panel of Table 3, this criterion was defined by the (1) frequency of performance required to be effective; (2) feasibility of the behavior (e.g., single versus multiple steps, required negotiation or engagement of others); and (3) availability and accessibility of required materials (e.g., a brush to scrub a water-storage container). The locus of control was also considered here and whether an individual could carry out the behavior independently. As was done in the evidence review, each behavior's ease of use/amenability to change was rated as “high,” “medium,” or “low” (excepting “feasibility,” which was ranked as “easy,” “medium,” or “complex”). To evaluate each behavior against this programmatic criterion, a consensus-building approach was implemented at meetings of the USAID Partners Zika SBC Technical Working Group and through consultations with technical experts. The SBC Technical Working Group aims to support collective SBC efforts by creating a forum to coordinate, share, and discuss challenges, solutions, and best practices for Zika prevention and promote evidence-based SBC practices. Members include USAID, UNICEF, and implementing partners working directly with households and communities in the region. Implementing partners are NGOs receiving USAID funding for the implementation of the Zika response.

To summarize, the prioritization process took place over 7 months between October 2017 and April 2018. More than 30 variants of behaviors and messages were identified and narrowed down to 7 through extensive discussion with the technical working group and input from technical experts to reach an initial consensus. To narrow down to these 7 behaviors, we used a combination of expert opinion, initial scan of available evidence, and factors such as whether USAID was supporting the behavior by procuring the materials necessary. Two of the final 7 behaviors were considered enabling behaviors and recommended but not included in the evidence review, given the substantial existing evidence on these behaviors. Five behaviors were explored in a full literature review to ascertain their relative effectiveness. During the literature review phase, partners provided additional gray literature where applicable. The full findings were presented at a subsequent SBC Technical Working Group meeting to ensure a consensus on the findings. The initial purpose of presenting the findings was to further narrow down the prioritized behaviors, but after partners expressed significant pushback, the full list of 7 behaviors were agreed upon. Any feedback from implementing partners regarding how behaviors were carried out, contextual challenges, or unpublished effectiveness findings was integrated into the final recommendations, and the results were disseminated to all USAID implementing partners.

More than 30 behaviors and messages were narrowed down to 7 after reaching consensus within the Zika SBC Technical Working Group.

## RESULTS

The findings and conclusions for Zika SBC programming drawn from this process are presented below for each of the 5 preventive behaviors reviewed, as well as the 2 enabling behaviors.

### Personal Protective Behaviors

**Applying mosquito repellent:** Application of mosquito repellent is highly efficacious in preventing mosquito bites, and thus the potential of vector transmission of Zika to an individual. This behavior is within the control of pregnant women and male partners of pregnant women. It is recommended that users be thoroughly counseled on proper product application. Women intending to become pregnant should also consider using repellent.

Applying effective mosquito repellant properly and consistently is highly efficacious in preventing mosquito bites and potential transmission of Zika.

Applying mosquito repellent (DEET, picaridin, IR3535, or lemon eucalyptus oil) and using each product as directed is a highly effective method in preventing mosquito bites and reducing risk of Zika transmission through mosquito bites. DEET is considered the gold standard repellent, showing greater than 95% efficacy in preventing mosquito bites for 5–11 hours.10 USAID and CDC approved 3 additional repellents (picaridin, IR3535, lemon eucalyptus oil) based on evidence suggesting they have an efficacy comparable to DEET.11 These 4 repellents are the only ones for which evidence of effectiveness has been recorded. The use of these repellents was also considered safe for use during pregnancy.^11^^-^^13^ We did not find program or intervention studies that assessed the impact of mosquito repellent on arbovirus disease transmission, but deduced a strong likelihood of effectiveness because it prevents mosquito bites. Concerning the third criterion—“easy to do and amenable to change”—repellent must be applied frequently (multiple times per day) to be effective, particularly if the person is sweating, swimming, or changing clothes.^10^^,^^14^ The use of repellent was considered to have medium feasibility because individuals decide whether to use repellent and therefore have control; however, those who have low literacy or limited access to trained antenatal care or pharmacies may have a disadvantage in following the written package instructions. Mosquito repellents are sold on the market in most countries in LAC and generally available in these settings. Contextual information regarding repellents was reported from partners working in the field with local knowledge. USAID also has procured repellents for distribution at antenatal care clinics in a few select countries. The price may be a barrier, particularly for low-income households.

**Using condoms to prevent sexual transmission:** Condom use to prevent sexual transmission of Zika is highly efficacious, but sexual transmission may be a small portion of overall transmission. This behavior should be prioritized for pregnant women and their partners because pregnant women are at risk for negative pregnancy outcomes.

Using condoms to prevent sexual transmission of Zika is highly efficacious and important during pregnancy to prevent congenital Zika syndrome.

There is evidence that Zika is transmitted sexually.^15^^-^^17^ According to our evidence review, condoms are highly effective in preventing sexually transmitted infections.18 Condom use is the only behavior that can prevent sexual transmission of Zika to sexually active women who may become pregnant or already are pregnant. Although statistical modeling suggests that sexual transmission of Zika is only 4%–5% of total transmission in the general population,^19^^-^^20^ the attributable risk of exposure among sexually active women may be twice as high.21 This increased risk of exposure, combined with the severity of outcomes in pregnancy, led to the identification of pregnant women and their partners as a target population for messaging about condom use. We found that condom-use behavior had a mixed amenability to change for 3 reasons: first, condoms must be worn consistently and correctly to be effective (on the basis of findings from the sexually transmitted infection literature), including throughout pregnancy^17^^,^^22^; second, the behavior is complex, requiring negotiation between partners^23^^-^^26^; and third, since condom use is not considered a normative behavior during pregnancy, it may be challenging to promote and adopt.^24^ Condoms are widely available in pharmacies and health centers in LAC, but access may be limited for women of low income.^27^

### Household and Community Vector Control

**Regularly removing unintentional standing water both inside and outside the house:** This is a potentially efficacious behavior to reduce mosquito populations and reduce the potential for individual- and population-level risk of Zika transmission. Promotion of the behavior must be accompanied by specific, focused instructions that target the highest density breeding sites and be conducted weekly in homes and communal areas to be effective. Efficacy is highest in areas with strong community engagement, including active mosquito searches in homes and communities and awareness of the mosquito life cycle.

Ongoing community clean-up campaigns to remove unintentional standing water can reduce the mosquito population and thus reduce potential Zika transmission.

According to the evidence, performing this behavior is highly efficacious in reducing the adult *Aedes* mosquito population. One study found a greater than 70% reduction in the adult mosquito population following a very strict intervention, involving community campaigns and visits from trained volunteers, to remove stagnant water.^28^ However, individuals and households may find it challenging to perform this behavior correctly and consistently. To have an impact on the *Aedes* mosquito population, and thus Zika transmission, it requires an ongoing, collective effort,^28^^-^^29^ including households as well as common areas, such as schools, clinics, cemeteries, and others. To maximize the potential impact, efforts need to focus on the highest-density mosquito-breeding sites as identified by entomological data collection.^30^^-^^32^ General clean-up campaigns in which communities receive information to clean their yards or communal areas without specificity on targets for removal often are only effective if they target the most productive breeding sites.^33^ In addition, these kinds of interventions are challenging to measure since clean-up campaigns are often conducted in conjunction with other interventions, and studies do not isolate the effect of any one of them.^33^ Despite these concerns, when instructions are clear and focused, regular removal of standing water is relatively easy to do and amenable to change; special materials are unnecessary in most cases. However, the feasibility is somewhat complex; the targeting of breeding sites should depend on how productive they are (to identify the highest-density breeding sites), and those sites are often either difficult to access (e.g., in storm drains) or located in communal areas requiring collective effort and engagement to attempt (e.g., in schools or construction sites). In addition, this behavior requires weekly action, based on the life cycle of *Aedes* mosquitoes.^34^

**Covering water storage containers at all times with a tight-fitting cover that does not warp or touch the water:** Covering long-term water storage containers has moderate potential efficacy in reducing breeding sites if a tight-fitting, long-lasting lid is available. Covering short-term water storage containers has less potential efficacy, as frequent lid use can result in wear and tear and render the lids ineffective or counterproductive.

Covering long-term household water storage containers with tight-fitting, long-lasting lids can prevent mosquito breeding sites.

The focus of this behavior is on long-term storage items such as barrels or other large household water-storage containers used less than once per week. A small number of studies suggest that the correct use of lids is associated with a significant reduction in pupal infestation if the containers are used infrequently.^35^ However, correct use and adequate lids are critical; if the lid is broken or touches the water in the container, the lid itself can spawn a breeding site for *Aedes* mosquitoes.^34^ The beneficial effect of correctly using a lid is mixed or even reversed if the water-storage container is used very often, constantly opened and closed, or often left open.^34^ This behavior, when done correctly, may reduce transmission at the population level, but in most published evidence, it is combined with community mobilization and cleaning of containers, making it challenging to isolate its effect.^35^ The behavior itself is relatively easy to implement. For long-term storage containers (from which water is accessed infrequently), the frequency of removing lids is low^34^ and relatively feasible, assuming the lids are used correctly.^34^ However, as research is ongoing to determine what type of lids are the most effective, access to proper lids was rated low. For short-term water storage, the frequent opening and closing of lids and additional requirement of monitoring the quality of the cover reduces ease of implementation, and therefore, its effectiveness. Long-term water storage containers coupled with correct use of tight-fitting, long-lasting lids, may enable this behavior to have moderate potential efficacy in reducing *Aedes* breeding sites.

**Eliminating mosquito eggs from water-storage container walls weekly:** Thorough cleaning of water-storage containers can remove mosquito eggs, significantly reducing the population and, thus, Zika transmission, but easy access to effective materials cannot be assumed.

*Aedes aegypti* mosquitoes lay their eggs in water storage containers, such as washbasins and metal drums, located inside or outside the house, increasing the risk of transmission of diseases such as Zika to households. As a result, cleaning containers is often recommended, but historically a lack of specific instruction has led to mixed results. For example, the World Health Organization recommends scrubbing containers with a brush, but does not mention whether a cleaning solution (such as bleach or detergent) should be applied, and cites studies that do not isolate the effect of cleaning from other behaviors (such as using lids).^36^ Research from the early 1990s reported manual cleaning of containers was ineffective in removing mosquito eggs, but it is unclear exactly how the containers were being cleaned and if eggs were targeted incorrectly.^37^^-^^38^ Since that time, several new methods have been developed. In our review, we judged 4 methods to be effective based on available efficacy evidence and consultations with entomologists with field experiences in the region (listed here in decreasing order of effectiveness).

1. The Untadita method, tested in a randomized controlled trial,^39^ was found to be more effective than scrubbing alone. In this method, a specific bleach and non-ammonia detergent mixture is applied to container walls that are then scrubbed with a brush and rinsed out after 10 minutes.^39^ Although this method has been promoted, there have been concerns in the field regarding potential toxicity of mixing non-recommended types of detergent with chlorine and the need to fully empty the container, which is challenging in water-scarce areas. In one study, 82% of surveyed households stored water and cited interruption of water services, poor water pressure, or cost-saving concerns as reasons for not wanting to empty their water-storage containers.^30^
2. The second method, developed in the Negociación de Prácticas Mejoradas trial,^40^ requires applying bleach to water-storage container walls without being emptied if they are partially filled.^40^ No field-based results on effectiveness or the intervention at scale were available, but small and experimental tests suggested positive ovicidal results.^41^
3. If the first 2 cleaning methods are not possible to carry out due to lack of bleach, the third technique of cleaning the walls of the container with detergent alone (using a brush, if available) should be implemented. This technique requires fully emptying the container.
4. Lastly, scrubbing the container walls with a brush (only) is recommended if neither detergent nor bleach is available.

### Enabling Behaviors

**Seeking antenatal care.** Seeking antenatal care is known to contribute to healthy pregnancies. In this setting, seeking antenatal care enables counseling on Zika prevention by trained health care providers, allowing for early diagnosis and treatment, as well as access to information about effective protective measures to reduce the risk of transmission of Zika from mother to child.

**Using contraception voluntarily.** Seeking family planning (for those not intending to get pregnant) is also a critical behavior, linked to prevention of sexually transmitted infections and prevention of sexual transmission of the Zika virus. Both of these behaviors—seeking family planning and antenatal care—are routinely promoted for adoption of healthy behaviors among pregnant women and women of reproductive age.

### Results Dissemination

The results of the literature review and the consultative process discussed in this article helped to identify behaviors with the greatest promise for preventing the acquisition and transmission of Zika. A critical part of this process was the consensus-building process with partners, ensuring input from those working on the ground. The SBC Technical Working Group engaged partners throughout the process. Partners requested focusing on behaviors that families and communities could do themselves (“locus of control” at the household). Points of contention often centered around behaviors that were being promoted already and were perceived by partners to be effective but had mixed evidence. For example, covering water storage containers was found to effective in the literature only for long-term water storage containers, but ultimately short-term containers were included in the guidance based on conversations with partners. This was mainly because partners perceived this behavior (covering water storage containers with a lid) to be effective based on this behavior being implemented already and being received positively. However, for other behaviors, for example use of bed nets, we clarified that although these are effective for malaria, they are not effective for Zika, and this was ultimately agreed upon and the guidance accepted. Through the process, partners were reminded that choices had to be made to prioritize key behaviors; although all of them were potentially effective, we were looking for relative effectiveness to prioritize the most effective ones and focus on them. Where there was pushback, we asked for field data to inform the decision to modify the final guidance.

These results were summarized in the Zika Prevention Behavior Matrix, a document widely disseminated through the Zika Communication Network (ZCN), a platform for sharing Zika-related resources and media products in the LAC region. A Technical Specifications Content Guide (a companion to the matrix) was also developed to detail the evidence-based technical requirements and steps to follow for each of the 7 behaviors (described here in Table 1, and in more detail in the Technical Specifications Content Guide) to reduce transmission. The guide was made available on the ZCN website to guide implementing partners in developing SBC content. Both documents, available in English and Spanish, guide prevention messages and prioritize calls to action to harmonize partner efforts and clarify specific messages to families, communities, and health care providers targeted by SBC programs for prioritized behaviors. The documents will be available to the public via an interactive digital platform that will guide users through the evidence, messaging, and technical specifications in a user-friendly way. Because *Aedes aegypti* vector control and mosquito bite prevention behaviors are included in this resource, partners working on dengue and chikungunya can use it. The documents will be continually updated as new developments emerge, reflecting input from implementing partners, to ensure that the materials address realities on the ground. For example, based on input from the field, guidance on correct disposal of repellents was recently added.

Additional resources have been developed by Breakthrough ACTION to support the promotion of the prioritized behaviors with the needed specificity described in the Technical Specifications Content Guide. SBC program teams in the field can use these tools to adapt their efforts to the latest findings and recommendations. A job aid has been developed to guide outreach workers and volunteers during household visits to better target audience segmentation for behaviors to maximize the uptake of the recommendations in the Zika Prevention Behavior Matrix. To facilitate effective use of the job aid and messaging around the prevention behaviors, a training-of-trainers curriculum on interpersonal communication skills for outreach workers has also been developed. This curriculum has been used to train health promoters, volunteers, and field technicians in 5 LAC countries and adapted for context-specific variation with USAID implementing partners and/or Ministry of Health personnel. Both the job aid (in English and Spanish) and curriculum are available on the ZCN. Country-specific adaptations to the job aid, such as including language for dengue and chikungunya prevention, are also available on the ZCN.

## DISCUSSION

The conditions brought on by climate change, international travel, urbanization, deforestation and other global and regional trends may result in new emerging diseases, as well as the spread of existing diseases to previously unexposed populations.^42^ Ministries of Health, international organizations, and NGOs must respond rapidly to outbreaks by coordinating an effective public health response. Under these circumstances, institutions rarely have sufficient data to guide SBC messaging for the most effective preventive actions for individuals and communities and may be forced to launch interventions or programs at the same time data are being gathered, assessed, and synthesized. This situation often leads to a lack of cohesion in the promoted behaviors and SBC messages. The behavior prioritization process documented in this article was developed to help USAID implementing partners identify focal behaviors for prevention to harmonize SBC programming efforts for greater impact. The process combined available evidence and a consensus-building approach to allow for adaptation based on local context. The consensus-building approach was critical for selecting behaviors and necessitated coordination across all stakeholders involved in the response across disciplines (e.g., public health, entomology, medical, and other technical area experts) and across response partners (e.g., those involved in mass media, service delivery, and community engagement). Types of behaviors were selected based on the transmission dynamics of the disease; Zika is transmitted by vector (mosquito) and sexually, highlighting preventive behaviors to consider. Behavior change is complex, and each behavior is comprised of many different behaviors and decisions that have to be addressed to successfully change. Each behavior we identified was rated against 3 criteria, 2 related to supporting evidence and 1 related to feasibility and amenability of the behavior to change.

The behavior prioritization process was developed to identify focal behaviors for prevention to harmonize SBC programming efforts for greater impact.

The first 2 criteria of the process assessed the state of the evidence available for selecting key behaviors to promote. The availability and quality of evidence depends on how long the disease has been around and whether it is occurring in a new region or sub-population. For example, in the 2002 severe acute respiratory syndrome outbreak, there was no evidence as the world was contending against a newly encountered disease. During the West Africa Ebola outbreak, researchers were attempting to understand the behaviors to target to interrupt transmission, while programmers were developing prevention and treatment programs and messages in real-time; research and evidence generation came later. In the case of Zika, the disease was previously known in east Asia and the Pacific but was entirely new to LAC. However, other *Aedes aegypti* mosquito viruses were prevalent in the region, specifically, dengue, which had been circulating in the region for more than 50 years, and chikungunya had emerged in 2013. Thus, information regarding best practices to prevent transmission could be gleaned from previous mosquito control and behavior change programming from LAC and southeast Asia and could be adapted to Zika prevention efforts.

Two unique characteristics of Zika presented themselves when applying the evidence. First, unlike dengue or chikungunya, Zika is mostly asymptomatic or presents mild symptoms; LAC is the first setting where it has been widely associated with adverse pregnancy outcomes (and Guillain-Barré syndrome). Second, it was found that Zika can be transmitted sexually, but other arboviruses, such as dengue and chikungunya, cannot. Although we were able to adapt an extensive evidence base for vector control of the *Aedes aegypti* population, there were little to no published data available regarding sexual transmission of Zika or its impact on pregnancy outcomes. Sexual transmission and the link with congenital malformations meant we needed to consider specific behaviors and messaging directed to pregnant women and women of reproductive age. To address this gap, evidence was gleaned and adapted from the literature on sexually transmitted infections and from research findings as they emerged from the field (before publication).

The third criteria of the process comprised a set of 3 components related to behavior amenability to change in the LAC setting. This step was an important contextual step because the effectiveness of a behavior is largely dependent on whether people are able and willing to perform it and was additionally complicated because Zika was new to the region. Because the behavior prioritization process was started after a year of Zika response, local partners had field experience to advise on which priority behaviors were feasible to implement and which materials were available and affordable. A consensus-building approach with USAID and its implementing partners was developed to categorize the relevant behaviors by criteria. Although the evidence review highlighted behaviors with potential to prevent diseases with the same modes of transmission or reduction of the same vector population as Zika, it was critical for each focal behavior to be assessed through the lens of this third criteria to ensure partner buy-in—that recommendations were realistic and reflected the context on the ground. The result of the process was a set of priority behaviors based on a combination of research evidence and context from partners with local knowledge.

The result of the process was a set of priority behaviors based on a combination of research evidence and context from partners with local knowledge.

The behavior prioritization process was developed to streamline the Zika response but began mid-implementation, by which time many partners were already deploying various behavior change recommendations. Any suggested changes to SBC programming that stemmed from the findings of this necessitated midcourse corrections. All 5 behaviors were prioritized by the USAID Zika response across 20 countries, and included interventions such as regional mass media campaigns, household and school-based SBC activities, household visits by vector control technicians, community fairs, and health care provider counseling. Several key products were developed and made available to implementing partners to facilitate the incorporation of the findings of the process, including a list of priority behaviors and detailed guidance on how to correctly perform each behavior for maximum effectiveness (through a Behavior Matrix [Supplement 1], Technical Specifications Content Guide [Supplement 2], and a job aid). These products were useful for SBC programs to reconfigure and refine their messages as they continued to implement their SBC interventions. Many partners reported that the guidance provided a basis to focus their limited remaining resources and to attain the needed specificity for each behavior to be effectively implemented. The iterative, collaborative process of defining behaviors across all stakeholders was critical to ensuring a more harmonized and feasible response. This process strives to incorporate evidence where it is available and was refined as the work developed, allowing it to be responsive to new evidence and contextualized based on input and expertise from those on the ground. This process can identify and select behaviors with the most potential to reduce transmission, is designed to be adapted to local contexts, and is flexible and based on consensus building with local and international stakeholders. Our experience developing this process provides a potential model for future public health emergencies, as it highlights a way forward in prioritizing behaviors in evidence in situations where direct evidence is limited or absent, time is constrained, and there are many key stakeholders.

## Supplementary Material

19-00188-Pinchoff-Supplement1.pdf

19-00188-Pinchoff-Supplement2.pdf

## Notes

**En español**

**Prevención del Zika en América Latina y el Caribe Priorización de comportamientos clave basado en la evidencia y su aplicabilidad a futuras respuestas de emergencia de salud pública**

En el propósito de maximizar el impacto de los esfuerzos de prevención del Zika, se estableció un proceso de priorización para los programas de cambio social y de comportamiento en base a una combinación de evidencia sólida y proveniente de investigaciones y experiencia programática. Los comportamientos priorizados fueron: aplicación de repelente de mosquitos, uso de condones, eliminación de agua acumulada alrededor de la casa y comunidad, cubrir los recipientes de almacenamiento de agua, fregar las paredes de los recipientes de almacenamiento de agua, asistencia a las consultas de atención prenatal, y la busqueda de consejería sobre planificación familiar si no planea un embarazo.

**Resumen**

Desde el brote del Zika de 2015 en América Latina y el Caribe, se venian promoviendo una gran cantidad de mensajes de cambio de comportamiento para reducir la transmisión del Zika. Un año después de que la Agencia de los Estados Unidos para el Desarrollo Internacional (USAID) iniciara su respuesta al Zika, se encontró que se venían promoviendo más de 30 variantes de conductas preventivas. Esta situación planteó un desafió a los esfuerzos de los programas de cambio social y de comportamiento (CSC) que requieren de una respuesta coordinada y de común acuerdo sobre el conjunto de comportamientos a promover para lograr máyor efectividad. Para apoyar a los socios implementadores de USAID en la armonización de los esfuerzos de prevención para reducir la infección por Zika, se desarrolló un proceso basado en evidencia que identificara aquellos comportamientos con mayor potencial en reducir la infección y transmisión del Zika. Se recopiló una lista completa de comportamientos y se seleccionaron los más prometedores para luego llevar adelante una revisión de la evidencia presentada. La revisión incluyó búsquedas sistemáticas de palabras clave en Google Scholar, identificación de todos los artículos publicados entre 2012 y 2018 que fueran relevantes a las enfermedades transmitidas por el mosquito Aedes, y revisión de documentos pioneros y de literatura gris. Se examinaron una serie de artículos para determinar la efectividad potencial de cada comportamiento en prevenir la transmisión del Zika o en reducir la población de Aedes aegypti. Igualmente se desarrollaron criterios de evaluación para medir la facilidad con la que la población objetivo podría adoptar cada comportamiento, incluyendo: (1) frecuencia requerida; (2) viabilidad del comportamiento; y (3) accesibilidad y costo de los materiales necesarios en el contexto inmediato. Estos comportamientos se refinaron con los socios implementadores de USAID de la respuesta al Zika a través de un proceso de creación de consensos considerando factores contextuales. El resultado fué la selección de 7 comportamientos preventivos que según la evidencia presentaban el mayor potencial para lograr el impacto de los programas de CSC en la respuesta al Zika de USAID: (1) aplique repelente de mosquitos, (2) use condones durante el embarazo, (3) elimine agua acumulada alrededor de la casa y comunidad, (4) cubra los recipientes de almacenamiento de agua, (5) elimine los huevos de mosquito de los recipientes de agua, (6) busque atención prenatal, y (7) busque consejería en planificación familiar si no planea un embarazo. El presente Estudio de Caso documenta un proceso flexible que puede ser adaptado para llevar adelante ejercicios de priorización de comportamientos cuando se cuenta con una evidencia limitada, como bien sucede en muchas de las respuestas de emergencia.

**Peer Reviewed**

**Funding:** Breakthrough-RESEARCH is made possible by the generous support of the American people through the United States Agency for International Development (USAID) under the terms of cooperative agreement no. AID-OAA-A-17-00018.
