# Supplementary material for: Evidence-Based Process for Prioritizing Positive Behaviors for Promotion: Zika Prevention in Latin America and the Caribbean and Applicability to Future Health Emergency Responses
Source: Glob Health Sci Pract. 2019 Sep 23;7(3):404–17. doi: 10.9745/GHSP-D-19-00188 (PMC6816817; doi:10.9745/GHSP-D-19-00188)
Supplement: 19-00188-Pinchoff-Supplement1.pdf [file 19-00188-Pinchoff-Supplement1.pdf]

Zika Prevention Behavior Matrix

USAID FROM THE AMERICAN PEOPLE

Breakthrough ACTION + RESEARCH FOR SOCIAL & BEHAVIOR CHANGE

Acknowledgements

Breakthrough ACTION and Breakthrough RESEARCH, with the United States Agency for International Development (USAID), would like to acknowledge UNICEF, PSI, Save the Children, IFRC, Abt Associates, MCDI, Global Communities, AMOS/SSI, CARE, the Pan American Development Fund, the Centers for Disease Control and Prevention, Tulane University, Population Council and the Johns Hopkins Center for Communication Programs for their technical expertise and participation in the process of identifying the behaviors with the most potential for Zika prevention.

This document is made possible by the generous support of the American people through the United States Agency for International Development (USAID). The contents are the responsibility of Breakthrough ACTION and Breakthrough RESEARCH and do not necessarily reflect the views of USAID or the United States Government.

Table of Contents

Introduction………………………………………………………………………………..…………… **4**
Zika Prevention Behavior Matrices…………………………………………………………….. **7**
Personal Protection Behaviors…………………………………………………………………………………… 7
Household and Community Vector Control Behaviors……………………………………………………... 9
Enabling Behaviors……………………………………………………………………………………………………... 12
References……..…… 14
Annex 1: Annotated Bibliography…………………………………………………………….. **16**
Annex 2: Behaviors Not Selected for Further Review…………………………….……... **30**

Zika Prevention Behavior Matrix

Introduction

PURPOSE

The purpose of the Zika Prevention Behavior Matrix is to focus Zika prevention efforts on the key behaviors that have the greatest potential to reduce Zika transmission and minimize negative pregnancy outcomes. During the first year of the USAID Zika response in Latin America and the Caribbean, USAID found that over 30 behaviors were being promoted. This large number of behaviors presents a challenge to the potential effectiveness of social and behavior change (SBC) efforts to prevent Zika at the household and community level. Lessons learned from behavioral science, marketing and adult learning have demonstrated that individuals need to hear a message multiple times before they are compelled to take action. It is therefore crucial for partners working in SBC for Zika prevention to promote a harmonized set of behaviors, to "speak with one voice" and increase the chances that these behaviors are adopted correctly. This conclusion prompted the need to determine which of the many behaviors currently promoted have the largest potential impact to reduce Zika transmission, in order to focus prevention efforts around a shorter, common set of most promising behaviors.

The Zika Prevention Behavior Matrix is the outcome of a review of supporting literature around the relative potential efficacy of various Zika prevention behaviors and a consultative process to determine the key prevention behaviors with the greatest potential impact. The matrix was created in partnership with USAID, UNICEF, and USAID implementing partners and serves as a reference document for partners in the Zika response.

PROCESS

USAID, Breakthrough ACTION and Breakthrough RESEARCH followed a multi-step process to identify and review relevant literature on Zika prevention behaviors. Since little research or data is available on the effect of preventive behaviors on Zika transmission, the team considered literature from dengue, chikungunya and other diseases transmitted by *Aedes aegypti* as proxies. Additionally, lessons learned and supporting evidence were captured from the extensive research in the fields of HIV, regarding condom use; of reproductive health, regarding family planning; and of maternal health, regarding prenatal care seeking.

The team made reasonable assumptions that literature indicating a reduction in *Aedes aegypti* breeding sites provided supporting evidence for the potential of reduced Zika transmission to humans, and as a result fewer negative pregnancy outcomes (e.g. cases of Congenital Zika Syndrome – CZS). Thus, while there is little direct evidence of the effects of interventions on Zika transmission, and existing data on preventive behaviors may not demonstrate causality, literature with proxy entomological indicators and indirect evidence support the evaluation of which behaviors potentially have the greatest prevention impact.

The process the team undertook can be organized into three different phases:

1. **Identification and categorization of Zika prevention behaviors currently promoted**
   - Based on a brief review of Zika communication materials in use, 30+ behaviors were being promoted across the USAID Zika response.
   - The team reviewed the range of Zika prevention behaviors being promoted and grouped them into three categories.

- Initial review of the literature on these behaviors informed the selection of the seven most promising behaviors for further review:
  - **Personal Protection**
    - Application of mosquito repellent (DEET, Picaridin, IR3535, or oil of lemon eucalyptus, only), using each product as directed, for duration of pregnancy, to reduce risk of Zika transmission through mosquito bites
    - Use of condoms to prevent sexual transmission of Zika in pregnancy.
  - **Household and Community Vector Control**
    - Regularly removing unintentional standing water both inside and outside of the house, and in communal areas.
    - Covering water storage containers at all times with a cover that is tight fitting and does not warp or touch the water.
    - Eliminating mosquito eggs from walls of water storage containers weekly.
  - **Enabling Behaviors -** behaviors that do not directly prevent Zika transmission and/or CZS, but facilitate an intervention that effectively contributes to their prevention
    - Seeking prenatal care to monitor pregnancy and discuss Zika risk and prevention.
    - Seeking counseling from a trained provider on modern family planning methods if not planning on getting pregnant.

2. **Literature Review**

This step involved a more in-depth review of evidence supporting the potential efficacy of each behavior to reduce Zika transmission and therefore minimize the risk of negative pregnancy outcomes from Zika (CZS).

Over 100 articles were reviewed through a rigorous search of Google Scholar (quantitative and qualitative data) as well as unpublished references and reports from partners. The review was mostly limited to articles published after 2012. The team developed an annotated bibliography (Annex 1) and summarized key findings for each behavior. The evidence for behaviors with insufficient efficacy data is described in Annex 2.

3. **Creating the Zika Prevention Behavior Matrix**

Based on the behaviors identified and the research review conducted, the team developed a matrix to examine each behavior for its potential to reduce Zika transmission and minimize negative pregnancy outcomes.

The team developed three criteria to analyze each behavior in terms of efficacy, effectiveness and feasibility. Color-coding was used to show the rating of each criteria according to the literature and field experience. At the bottom of each table there is a summary statement on the overall efficacy and feasibility of the behavior.

Zika Prevention Behavior Matrix

Criteria	Ratings	Definition
**1. Proven efficacy of the behavior**	High<br>Medium<br>Low	Do the findings from the literature demonstrate that the behavior is efficacious in a research setting? For instance, does the behavior have an effect on:<br>• reducing risk of Zika transmission<br>• reducing the risk of negative pregnancy outcomes<br>• reducing *Aedes* aegypti breeding sites
**2. Potential to reduce Zika transmission at population level**	High<br>Medium<br>Low	To what scale or degree can this behavior contribute to reduction in Zika transmission at the population level when implemented? "Biggest bang for the buck."
**3. Easy to do / Amenable to Change**		*Is the behavior easy to carry out?*
**a) Frequency required to be effective**	Low<br>Medium<br>High	How often does it need to be practiced?<br>• **Low**: monthly, one time only<br>• **Medium**: a few times a week, weekly<br>• **High:** multiple times a day, daily
**b) Feasibility of behavior**	Easy<br>Medium<br>Complex	How feasible is the process to practice the behavior effectively? Does it involve multiple steps? Does it require negotiation?
**c) Ease of access to materials required**	High<br>Medium<br>Low	Are materials accessible (availability and cost) to households?
**Summary**	Statement summarizing efficacy and feasibility of this behavior.	

The team linked the available literature findings against each of the criteria (as applicable). Drafts of the matrix were reviewed and discussed during the USAID Zika SBC Working Group meetings with partners. Subsequent feedback informed decisions around the rating of each behavior, especially for criteria #3, where collective experience in the field helped determine the feasibility of each behavior in practice.

The following pages show the seven behaviors organized by criteria, with the pertinent data, as well as color-coding, to indicate the potential efficacy and feasibility. The literature is footnoted for easy access to the list of references included in the endnote page. Additionally, there is an annotated bibliography of all the literature reviewed (Annex 1).

Zika Prevention Behavior Matrix

I. Personal Protection

Behavior 1: Application of mosquito repellent (DEET, Picaridin, IR3535, or oil of lemon eucalyptus, only), using each product as directed, for duration of pregnancy, to reduce risk of Zika transmission through mosquito bites.

Criteria	PW	Male partners of PW	Supporting Literature & Information
**1. Efficacy of the behavior to reduce the risk of Zika transmission** (High/Med/Low)	High	High	Efficacy rated high because:<br>• The majority of available research is on DEET, and shows >95% efficacy in preventing mosquito bites for 5-11 hours.[1] It is considered safe for use in pregnancy at concentrations of 30% or less.[2, 3]<br>• USAID and CDC consider three additional repellents (Picaridin, IR3535, oil of lemon eucalyptus) to be of comparable efficacy to DEET and recommended.[4]<br>• The high efficacy of these repellents to prevent mosquito bites when used properly has the potential to reduce the risk of vector-borne Zika transmission to pregnant women and thus, reduce risk of negative pregnancy outcomes.
**2. Potential to reduce Zika transmission at population level** (High/Med/Low)	Low	Low	Potential rated low because:<br>• While repellents reduce biting by *Aedes* mosquitoes, they do not kill or reduce the vector population. As such, repellent use does not reduce overall disease incidence or reduce transmission of Zika.
**3. Easy to do / Amenable to Change**			
**a) Frequency required to be effective** (High/Med/Low)	High	High	Frequency rated high because:<br>• Repellents must be applied multiple times a day to be effective.<br>• Repellents must be applied more frequently if the person is sweating, swimming, or changes clothes.[1,5]
**b) Feasibility of behavior** (Complex/Med/ Easy)	Medium	Medium	Feasibility rated medium because:<br>• Application of repellent is within the control of the individual.<br>• Understanding the specific application steps for each repellent may be complex, particularly for populations with low-literacy and low access to Zika counseling at ANC visits or by trained pharmacists.<br>• Repellent can be applied by the individual, so it may be easy to do for some. But for others, such as low-literate women, it may be harder to follow written instructions.
**c) Ease of access to materials required** (High/Med/Low)	Medium	Medium	Ease of access rated medium because:<br>• Repellents are available on the market in all USAID Zika response countries. USAID is also procuring repellents as part of the Zika response.<br>• The price of repellent may be a barrier, unless repellent is subsidized or available at no cost.
**Summary**	Application of mosquito repellent is highly efficacious in preventing mosquito bites, and thus the potential of vector transmission of Zika to an individual. This behavior is within the control of pregnant women and male partners of pregnant women. It is recommended that users be thoroughly counseled on proper application of the product. Women intending to become pregnant should also consider using repellent.		

Zika Prevention Behavior Matrix

Behavior 2: Use of condoms to prevent sexual transmission of Zika in pregnancy.

Criteria	PW	Male partners of PW	Supporting Literature & Information
**1. Efficacy of the behavior to prevent sexual transmission of Zika** (High/Med/Low)	High	High	Efficacy rated high because:<br>• There is no research on the efficacy of condoms as a barrier to prevent Zika transmission, but there is extensive literature on condom efficacy in preventing sexually transmitted infections (STIs).[6]<br>• This is the only known behavior to prevent sexual transmission of Zika to sexually active pregnant women and condoms are efficacious in preventing STIs.
**2. Potential to reduce Zika transmission at population level** (High/Med/Low)	Low	Low	Potential rated low because:<br>• Modeling studies found that sexual transmission rates of the Zika virus range from 4-5% of total transmission in the general population.[7, 8]<br>• A recent study assessing household level transmission found a two-fold association for sexual contact, so the attributable risk of exposure among pregnant women and WRA may be higher.[9]
**3. Easy to do / Amenable to Change**			
**a) Frequency required to be effective** (High/Med/Low)	High	High	Frequency rated high because:<br>• Evidence from STIs indicates that a condom must be used consistently and correctly to be effective. Thus, it is reasonable to extrapolate that condoms must be used consistently and correctly to prevent sexual transmission of Zika. [10-12]<br>• In order to reduce the risk of negative pregnancy outcomes related to Zika, condoms should be used in every sexual act for the duration of pregnancy.
**b) Feasibility of behavior** (Complex/ Med/Easy)	Complex	Complex	Feasibility rated complex because:<br>• Requires negotiation.[13-16]<br>• It is not considered a normative behavior during pregnancy.[13, 14]<br>• Consistent and correct condom use throughout the pregnancy may be difficult.
**c) Ease of access to materials required** (High/Med/Low)	Medium	Medium	Ease of access rated medium because:<br>• Condoms are usually accessible in pharmacies and/or MoHs in Central America, less so in the Caribbean (based on country partner feedback).<br>• Access may be more difficult for low income women
**Summary**	Condom use to prevent sexual transmission of Zika is highly efficacious, although sexual transmission may be a small portion of overall transmission. This behavior should be prioritized for pregnant women and their partners, since pregnant women are at risk for negative pregnancy outcomes.		

Zika Prevention Behavior Matrix

II. Household and Community Vector Control

Behavior 3: Regularly removing unintentional standing water both inside and outside of the house, and in communal areas.

<table>
<tr><th>Criteria</th><th>All populations</th><th>Supporting Literature & Information</th></tr>
<tr><td>1. Efficacy of the behavior to reduce breeding sites (High/Med/Low)</td><td>High</td><td>Efficacy rated high because:
• Multiple studies have found that removal of stagnant water is associated with reduction in adult mosquito populations. For example, one study found a more than 70% reduction.[17]</td></tr>
<tr><td>2. Potential to reduce Zika transmission at population level (High/Med/Low)</td><td>Medium</td><td>Potential rated medium because:
• Requires an ongoing, collective effort to impact the Aedes mosquito population.[17, 18] Includes areas such as schools, clinics, cemeteries, construction sites, etc.
• Efforts should focus on the highest density mosquito breeding sites based on entomological data, as well as household and community mosquito searches, to maximize the potential impact.[19-22] General clean-up is not as effective in eliminating the most important mosquito breeding sites and dilutes efforts.
• While this behavior can be efficacious if high density mosquito breeding sites are targeted frequently, it is often not carried out in a targeted manner.</td></tr>
<tr><td colspan="2">3. Easy to do / Amenable to Change</td><td></td></tr>
<tr><td>a) Frequency required to be effective (High/Med/Low)</td><td>Medium</td><td>Frequency rated medium because:
• Requires weekly action, based on mosquito life cycle.[23]</td></tr>
<tr><td>b) Feasibility of behavior (Complex/ Med/Easy)</td><td>Complex</td><td>Feasibility rated complex because:
• Favorable Aedes mosquito breeding sites are context specific. Unintentional standing water, such as rainwater, collects in many diverse areas (tires, bottles, cement pilas and wash basins) and not all are easily accessible (tree trunks, gutters, pot holes).
• Requires collective effort to remove unintentional standing water in communal areas, such as schools, clinics, cemeteries, and construction sites, and mapping of areas in the community where water inadvertently collects. This requires engagement with local businesses and households, and a community commitment.</td></tr>
<tr><td>c) Ease of access to materials required (High/Med/Low)</td><td>High</td><td>Ease of access rated high because:
• No materials needed in most cases.</td></tr>
<tr><td colspan="3"></td></tr>
<tr><td>Summary</td><td colspan="2">This is a potentially efficacious behavior to reduce mosquito populations, and thus reduce the potential for individual and population-level risk of Zika transmission. Promotion of the behavior must be accompanied by specific, focused instructions that target the highest density breeding sites and be conducted weekly in homes and communal areas in order to be effective. Efficacy is highest in areas where there is strong community engagement, including active mosquito searches in homes and communities, and awareness of the mosquito life cycle.</td></tr>
</table>

Zika Prevention Behavior Matrix

Behavior 4: Covering water storage containers at all times with a cover that is tight fitting, and does not warp or touch the water.

Criteria	All populations		Supporting Literature & Information
	Long-term Water Storage	Short-term Water Storage	
**1. Efficacy of the behavior to reduce breeding sites** (High/Med/Low)	Medium	Low	Efficacy of **long-term water storage** (those used less than once a week) rated medium because:<br>• Where water is used infrequently, a small number of studies suggest correct use of lids is associated with a significant reduction in pupal infestation.[24]<br>• Correct use is imperative – if the container lid is broken or dips in the water, it can become a breeding site itself.[23]<br>• For long-term water storage, covers are not used or opened frequently, thus they suffer less wear and tear.<br>Efficacy of **short-term water storage** (those used multiple times a day or several times a week) rated low because:<br>• The data shows lids can be effective at reducing pupal infestation if used correctly on large containers, but shows mixed or even reverse effect on frequently used containers.[23]
**2. Potential to reduce Zika transmission at population level** (High/Med/Low)	Medium	Low	Potential of **long-term water storage** rated medium because:<br>• When combined with community mobilization and scrubbing containers, covering containers was a component of a highly effective intervention that reduced entomological indices.[24]<br>• Effectiveness requires that tight-fitting lids are available and used consistently and correctly.[25]<br>Potential of **short-term water storage** rated low because:<br>• Effect of covering is diminished or potentially reversed for containers used frequently.[26]<br>• Some containers, like cement pilas and wash basins, are challenging to cover completely.
**3. Easy to do / Amenable to Change**			
**a) Frequency required to be effective** (High/Med/Low)	Low	High	• Frequency is low for **long-term water storage** containers (those used less than once a week).[23]<br>• Frequency is high for **short-term water storage** containers (those used multiple times a day or several times a week).
**b) Feasibility of behavior** (Complex/ Med/Easy)	Medium	Complex	• While covering containers may seem simple, the correct behavior is complex to implement, especially for short-term water storage. This depends on access to the correct type of cover, which needs to be used properly, carefully, and consistently.[23]<br>• Lids require replacement if they become warped or broken and require regular monitoring that the cover has not inadvertently created breeding sites,[25] especially for short-term water storage containers.
**c) Ease of access to materials required** (High/Med/Low)	Low	Low	• Effective lids may not be widely available (depending on context).
**Summary**	Covering long-term water storage containers has moderate potential efficacy in reducing breeding sites if a tight fitting, long-lasting lid is available. Covering short term water storage containers has less potential efficacy, as frequent lid use can result in wear and tear, and render the lids ineffective or counterproductive.		

Zika Prevention Behavior Matrix

Behavior 5: Eliminating mosquito eggs from walls of water storage containers weekly.

Criteria	All populations	Supporting Literature & Information
**1. Efficacy of the behavior to reduce breeding sites** (High/Med/Low)	High	Efficacy rated high because:<br>• Studies have shown that containers washed monthly or never were 2-4 times more likely to be infested than those washed weekly.(27, 28, 29)<br>• In comparison to general cleaning, the Untadita method (5-step process with chlorine bleach and household detergent) was found to be more effective at reducing infestation in a randomized trial.(30)
**2. Potential to reduce Zika transmission at population level** (High/Med/Low)	High	Potential rated high because:<br>• According to modeling data, elimination of mosquito breeding in water containers could reduce pupal population production by approximately 1/3, leading to reduced adult mosquito population and thus, a reduced risk of Zika transmission.(26)<br>• When combined with community mobilization and covering containers, scrubbing containers was also a component of highly effective intervention that reduced entomological indices.(24)
**3. Easy to do / Amenable to Change**		
**a) Frequency required to be effective** (High/Med/Low)	Medium	Frequency rated medium because:<br>• Requires action a minimum of once per week, according to vector control experts (ZAP) and literature(27) on efficacy of this behavior.
**b) Feasibility of behavior** (Complex/Med/ Easy)	Complex	Feasibility rated complex because:<br>• Requires a multi-step process with various materials (detergent, brushes), and there may be resistance to completely draining containers on a weekly basis due to irregular or expensive water supply.(30) Therefore, this weekly behavior may be most amenable to carry out for short-term water storage containers as opposed to long-term water storage containers.<br>• Ensuring the removal of eggs attached to walls is challenging (eggs may not always be visible against container wall and the scrubbing technique matters).(30)
**c) Ease of access to materials required** (High/Med/Low)	High	Ease of access rated high because:<br>• Household detergent and brushes are generally accessible and some USAID partners are providing brushes to households.
**Summary**	Scrubbing walls of water storage containers weekly is efficacious in removing mosquito eggs and can thus reduce the potential for individual and population-level risk of Zika transmission. However, the specific cleaning steps that eliminate mosquito eggs must be explicitly described.	

Zika Prevention Behavior Matrix

III. Enabling Behaviors – behaviors that do not directly prevent Zika transmission and/or CZS, but facilitate an intervention that effectively contributes to their prevention.

Behavior 6: Seeking prenatal care to monitor pregnancy and discuss Zika risk and prevention.

Criteria	Pregnant women	Partners of PW	Supporting Literature & Information
**1. Efficacy of the behavior to prevent negative pregnancy outcomes** (High/Med/Low)	High	High	Efficacy rated high because:<br>• Consistent prenatal care is known to contribute to healthy pregnancies.
**2. Potential to reduce Zika transmission at population level** (High/Med/Low)	n/a	n/a	
**3. Easy to do / Amenable to Change**			
**a) Frequency required to be effective** (High/Med/Low)	Low	Low	Frequency rated low because:<br>• Regular prenatal care is recommended and exact frequency depends on local protocols (see MOH and WHO guidelines).
**b) Feasibility of behavior** (Complex/Med/ Easy)	Medium	Medium	Feasibility rated medium because:<br>• Context specific, depending on distance, availability of prenatal care and norms around care-seeking.<br>• May require negotiation with family to support clinic visit.
**c) Ease of access to materials required** (High/Med/Low)	Medium	Medium	Ease of access rated medium because:<br>• Context specific, such as clinic fees and transportation.
**Summary**	Seeking prenatal care enables counseling on Zika prevention by providers, which can increase the chances of pregnant women taking protective measures and reduce the risk of vertical transmission of Zika from mother to child.		

Zika Prevention Behavior Matrix

Behavior 7: Seeking counseling from a trained provider on modern family planning methods if not planning on getting pregnant.

Criteria	WRA	Male Partners of WRA	Supporting Literature & Information
**1. Efficacy of the behavior to prevent negative pregnancy outcomes** (High/Med/Low)	High	High	Efficacy rated high because:<br>• Voluntary adoption of a modern family planning method has high potential to reduce vertical transmission of Zika from mother to child and therefore risk of CZS
**2. Potential to reduce Zika transmission at population level** (High/Med/Low)	n/a	n/a	
**3. Easy to do / Amenable to Change**			
**a) Frequency required to be effective** (High/Med/Low)	Medium	Medium	Frequency rated medium because:<br>• Depends on the FP method used. Some methods require daily action, while others require action monthly or every few years.
**b) Feasibility of behavior** (Complex/Med/ Easy)	Medium	Medium	Feasibility rated medium because:<br>• Depends on method – some require a daily pill, others require injection on a quarterly basis, others require medical insertion.<br>• May require negotiation with male partner.
**c) Ease of access to materials required** (High/Med/Low)	Medium	Medium	Ease of access rated medium because:<br>• Family planning is widely practiced, though there is not always access to a wide range of methods, and access is challenging for certain groups, such as adolescents.
**Summary**	Family planning use (for those not intending on getting pregnant) is directly linked to reducing the risk of vertical transmission of Zika. Family planning counseling should take place through a trained health provider.		

References

1. Lupi E, Hatz C, Schlagenhauf P. The efficacy of repellents against Aedes, Anopheles, Culex and Ixodes spp. - a literature review. Travel medicine and infectious disease. 2013;11(6):374-411.
2. Paumgartten F. Mosquito repellents, effectiveness in preventing diseases and safety during pregnancy. Vigil Sanit Debat. 2016;4(2):97-104.
3. Wylie BJ, Hauptman M, Woolf AD, Goldman RH. Insect Repellents During Pregnancy in the Era of the Zika Virus. Obstet Gynecol. 2016;128(5):1111-5.
4. Control CfD. Avoid Mosquito Bites | Features | CDC 2018 [Available from: https://www.cdc.gov/features/StopMosquitoes/.
5. Wong LP, AbuBakar S. Health beliefs and practices related to dengue fever: a focus group study. PLoS Neglected Tropical Disease. 2013;7(7):e2310.
6. Carvalho NS, Brazil FUoPDoGaOCP, Carvalho BF, Brazil PCUoPCP, Dóris B, Brazil PCUoPCP, et al. Zika virus and pregnancy: An overview. American Journal of Reproductive Immunology. 2018;77(2).
7. Coelho FC, Durovni B, Saraceni V, Lemos C, Codeco CT, Camargo S, et al. Higher incidence of Zika in adult women than adult men in Rio de Janeiro suggests a significant contribution of sexual transmission from men to women. International journal of infectious diseases : IJID : official publication of the International Society for Infectious Diseases. 2016;51:128-32.
8. Rao R, Gaw SL, Han CS, Platt LD, Silverman NS. Zika Risk and Pregnancy in Clinical Practice: Ongoing Experience as the Outbreak Evolves. Obstetrics and gynecology. 2017;129(6):1098-103.
9. Rosenberg ES, Doyle K, Munoz-Jordan J, Klein L, Adams L, Lozier M, et al., editors. Prevalence and incidence of Zika virus infection among household contacts of Zika patietns: Puerto Rico, 2016-2017. American Society for Tropical Medicine and Hygiene 2017; Baltimore, MD.
10. Control CfD. Sexual Transmission & Prevention | Zika Virus | CDC 2018 [updated 2018-01-31T07:28:02Z. Available from: https://www.cdc.gov/zika/prevention/sexual-transmission-prevention.html.
11. UNICEF. Risk Communication and Community Engagement for Zika Virus Prevention and Control 2016 [Available from: https://www.unicef.org/cbsc/files/Zika_Virus_Prevention_and_Control_UNICEF_English.pdf.
12. Organization WH. Prevention of sexual transmission of Zika virus 2016 [Available from: http://apps.who.int/iris/bitstream/10665/204421/1/WHO_ZIKV_MOC_16.1_eng.pdf.
13. Marteleto LJ, Weitzman A, Coutinho RZ, Valongueiro Alves S. Women's Reproductive Intentions and Behaviors during the Zika Epidemic in Brazil. Population and Development Review. 2018;43(2):199-227.
14. Zorrilla CD, Mosquera AM, Rabionet S, Rivera-Vinas J. HIV and ZIKA in Pregnancy: Parallel Stories and New Challenges. Obstetrics & gynecology international journal. 2016;5(6).
15. D'Angelo D, et al. Measures Taken to Prevent Zika Virus Infection During Pregnancy — Puerto Rico, 2016 | MMWR. 2017.
16. Fraiz LD, de Roche A, Mauro C, Catallozzi M, Zimet GD, Shapiro GK, et al. U.S. pregnant women's knowledge and attitudes about behavioral strategies and vaccines to prevent Zika acquisition. Vaccine. 2018;36(1):165-9.
17. Audraud M. A simple periodic-forced model for dengue fitted to incidence data in Singapore. - Abstract - Europe PMC. Mathematical Biosciences. 2013;244(1):22-4.
18. Alvarado-Castro V, Paredes-Solís S, Nava-Aguilera E, Morales-Pérez A, Alarcón-Morales L, Balderas-Vargas NA, et al. Assessing the effects of interventions for Aedes aegypti control: systematic review and meta-analysis of cluster randomised controlled trials. BMC Public Health. 2017;17(1).

19. Garcia-Betancourt T, Higuera-Mendieta DR, Gonzalez-Uribe C, Cortes S, Quintero J. Understanding Water Storage Practices of Urban Residents of an Endemic Dengue Area in Colombia: Perceptions, Rationale and Socio-Demographic Characteristics. PloS one. 2015;10(6):e0129054.
20. Quintero J, Brochero H, Manrique-Saide, Barrera-Pérez M, Basso C, Romero S, et al. Ecological, biological and social dimensions of dengue vector breeding in five urban settings of Latin America: a multi-country study. BMC Infectious Diseases. 2014;14(1):38.
21. Tran HP, Huynh TTT, Nguyen YT, Kutcher S, O'Rourke P, Marquart L, et al. Low Entomological Impact of New Water Supply Infrastructure in Southern Vietnam, with Reference to Dengue Vectors. Am J Trop Med Hyg. 2012;87(4):631-9.
22. Dom NC, Ahmad AH, Ishak AR, Ismail R. Assessing the Risk of Dengue Fever based on the Epidemiological, Environmental and Entomological Variables. Procedia - Social and Behavioral Sciences. 2013;105:183-94.
23. Phuanukoonnon S, Mueller I, Bryan JH. Effectiveness of dengue control practices in household water containers in Northeast Thailand. Tropical medicine & international health : TM & IH. 2005;10(8):755-63.
24. Morales-Pérez A, Nava-Aguilera E, Balanzar-Martínez A, Cortés-Guzmán AJ, Gasga-Salinas D, Rodríguez-Ramos IE, et al. Aedes aegypti breeding ecology in Guerrero: cross-sectional study of mosquito breeding sites from the baseline for the Camino Verde trial in Mexico. BMC Public Health. 2017;17(1).
25. Vannavong N, Seidu R, Stenstrom TA, Dada N, Overgaard HJ. Effects of socio-demographic characteristics and household water management on Aedes aegypti production in suburban and rural villages in Laos and Thailand. Parasites & vectors. 2017;10(1):170.
26. Hiscox A, Kaye A, Vongphayloth K, Banks I, Piffer M, Khammanithong P, et al. Risk factors for the presence of Aedes aegypti and Aedes albopictus in domestic water-holding containers in areas impacted by the Nam Theun 2 hydroelectric project, Laos. The American journal of tropical medicine and hygiene. 2013;88(6):1070-8.
27. Overgaard HJ, Olano VA, Jaramillo JF, Matiz MI, Sarmiento D, Stenstrom TA, et al. A cross-sectional survey of Aedes aegypti immature abundance in urban and rural household containers in central Colombia. Parasites & vectors. 2017;10(1):356.
28. Phuanukoonnon S, Mueller I, Bryan JH. Effectiveness of dengue control practices in household water containers in Northeast Thailand. Tropical medicine & international health : TM & IH. 2005;10(8):755-63.
29. Wanti W, Yudhastuti R, Yotopranoto S, Notobroto HB, Subekti S, Umniati SR. Container Positivity and Larva Distribution Based on the Container Characteristics. International Journal of Public Health Science. 2017;6(3):237-42
30. Fernandez EA, Leontsini E, Sherman C, Chan AS, Reyes CE, Lozano RC, et al. Trial of a community-based intervention to decrease infestation of Aedes aegypti mosquitoes in cement washbasins in El Progreso, Honduras. Acta tropica. 1998;70(2):171-83.

Annex 1: Annotated Bibliography

This annotated bibliography covers the following behaviors:

- **Behavior 1:** Application of mosquito repellent (DEET, Picaridin, IR3535, or oil of lemon eucalyptus, only), using each product as directed, for duration of pregnancy, to reduce risk of Zika transmission through mosquito bites.
- **Behavior 2:** Use of condoms to prevent sexual transmission of Zika in pregnancy.
- **Behavior 3:** Regularly removing unintentional standing water both inside and outside of the house, and in communal areas.
- **Behavior 4:** Covering water storage containers at all times with a cover that is tight fitting, and does not warp or touch the water.
- **Behavior 5:** Eliminating mosquito eggs from walls of water storage containers weekly.

Zika Prevention Behavior Matrix

Behavior 1: Application of mosquito repellent (DEET, Picaridin, IR3535, or oil of lemon eucalyptus, only), using each product as directed, for duration of pregnancy, to reduce risk of Zika transmission through mosquito bites.

Summary of the Literature Findings:

- **Efficacy:**
  - Efficacy of repellents is measured as either complete protection time (time between application and first two bites), percent repellency (number of mosquitoes landing or attempting to bite after application) or biting reduction (treatment to control comparison of bites) (Lupi, 2013).
  - Overall, DEET is considered the gold standard compared to other topical repellents on the market (Wong, 2016; Lupi, 2013) for preventing insect bites. On both *Aedes aegypti* and *Aedes albopictus* mosquito subspecies, DEET showed a period of >95% efficacy in preventing mosquito bites, lasting 5–11 hours (Lupi, 2013) under laboratory conditions. Using protection time criteria from WHO[1], DEET (25% concentration) met the recommended protection levels; it was found to protect for ≥ 6 hours in ≥ 90% of the people treated with DEET in a controlled, experimental setting (Uc-Puc, 2016). At 30% concentration the effect levels off, so most products are between 10 and 30% concentration.
  - Safety and toxicity data reviewed by EPA show low short-term toxicity and no significant health risks. Repellent containing DEET is currently recommended in pregnancy for safe use (30% concentration or less), and DEET use should not be combined with sunscreen. (Wylie 2016; Paumgartten 2016).
  - Three additional repellents (Picaridin, IR3535, and oil of lemon eucalyptus) were found to have comparable efficacy and safety according to CDC recommendations[2] and USAID guidelines (2016 Zika Control Programmatic PERSUAP).
- **Potential to reduce Zika transmission at population level:**
  - The potential for reducing Zika transmission is an inferential assessment from efficacy of preventing mosquito bites. There are no known studies that have effectively evaluated the population-level effectiveness of repellent use in reducing Zika transmission.
  - Insect repellents directly target the vector by reducing biting rates, but no studies have linked repellent use to infection risk for dengue (Bowman, 2016).
  - Use of repellent is a personal protective behavior aimed at preventing bites. According to a UNICEF review, such personal protective behaviors may "partially impact entomological indicators" (UNICEF, 2017).
- **Frequency required to be effective:**
  - The frequency of application required to be effective was determined from the literature and field experience.
  - Wearing repellent is highly effective in controlled settings, however, no studies have been identified of repellent use in Zika endemic settings. Studies do show that temperature/climate as well as activities that may dilute the repellent can vary the duration of effectiveness and need for reapplication (for example, swimming, sweating, washing, rubbing by clothes) (Lupi, 2013).
- **Feasibility of behavior:**
  - The assessment of feasibility was based on field experience.

---

[1] Based on protection time by WHO regulations: http://apps.who.int/iris/bitstream/10665/70072/1/WHO_HTM_NTD_WHOPES_2009.4_eng.pdf

[2] Centers for Disease Control and Prevention. Avoid Mosquito Bites. Link: https://www.cdc.gov/features/StopMosquitoes/

  - No field studies identified regarding repellent acceptability and uptake has been conducted in Zika endemic settings.
- **Ease of access to materials required:**
  - The assessment of ease of access was based on field experience and the procurement of repellent containing DEET by USAID as part of the Zika response.
  - No field studies identified regarding repellent availability and pricing in USAID Zika response settings. A market assessment conducted in Latin American countries with similar characteristics to the USAID Zika response countries found that repellents are available in formal channels, such as grocery stores, convenience stores and pharmacies. The assessment also found that many consumers cannot afford to constantly buy repellent (BCG, 2017).

List of Reviewed Literature:

1. Atif M, Azeem M, Sarwar M, Bashir A. Zika virus disease: a current review of the literature. Infection. 2016;44.
2. Fradin MS, Day JF. Comparative efficacy of insect repellents against mosquito bites. New England Journal of Medicine. 2002;347(1).
3. Karwowski M, Nelson J, Staples J, Fischer M, Fleming-Dutra K, Villanueva J, et al. Zika virus disease: a CDC update for pediatric healthcare providers. Pediatrics. 2016;137(5).
4. Leal W, Barbosa R, Zeng F, Faierstein G, Tan K, Paiva M, et al. Does Zika virus infection affect mosquito response to repellents? Scientific Reports. 2017.
5. Lupi E, Hatz C, Schlagenhauf P. The efficacy of repellents against Aedes, Anopheles, Culex and Ixodes spp. - a literature review. Travel medicine and infectious disease. 2013;11(6):374-411.
6. Miot H, Batistella R, Batista K, Volpato D, Augusto L, Madeira N, et al. Comparative study of the topical effectiveness of the Andiroba oil (Carapa guianensis) and DEET 50% as repellent for Aedes spp. Rev Inst Med trop S Paulo. 2004;46(5).
7. Neigh A, Martin J, Jolley J, Roy A, McCoy B. Global health Zika vector control: programmatic PERSUAP (Pesticide Evaluation Report & Safer Use Action Plan). Washington DC: USAID; 2016.
8. Nguyen N, Whitehorn J, Hue T, Thanh T, Xuan T, Xuan H, et al. Physicians, primary caregivers, and topical repellent: All under-utilised resources in stopping dengue virus transmission in affected households. PLOS Neglected Tropical Diseases. 2016;10(5).
9. Patel R, Shaeer K, Patel P, Garmaza A, Wiangkham K, Franks R, et al. EPA-Registered repellents for mosquitoes transmitting emerging viral disease. Pharmacotherapy. 2016;36(12)
10. Paumgartten F. Mosquito repellents, effectiveness in preventing diseases and safety during pregnancy. Vigil Sanit Debat. 2016;4(2):97-104.
11. Rodriguez S, Chung J, Gonzales K, Vulcan J, Li Y, Ahumada J, et al. Efficacy of some wearable devices compared with spray on insect repellents for the Yellow Fever mosquito, Aedes aegypti (L.) (Diptera: Culicidae). Journal of Insect Science. 2017;17(1).
12. Sathantriphop S, Kongmee M, Tainchum K, Suwansirisilp K, Sanguanpong U, Bangs M, et al. Comparison of field and laboratory-based tests for behavioral response of Aedes aegypti (Diptera: Culicidae) to repellents. Journal of Economic Entomology. 2015.
13. Stanczyk N, Brookfield J, Field L, Logan J. Aedes aegypti mosquitoes exhibit decreased repellency by DEET following previous exposure. PloS one. 2013;8(2).
14. Uc-Puc V, Herrera-Bojorquez J, Carmona-Carballo C, Che-Mendoza A, Medina-Barreiro A, Chable-Santos J, et al. Efectividad de repelentes comerciales disponibles contra el mosquito Aedes aegypti (L.) en Yucatan, Mexico. salud publica Mexico. 2016;58(4).
15. UNICEF. Review of independent evidence supporting vector control activities: Prevention of diseases spread by mosquitoes. New York NY: UNICEF; 2018.

16. USAID. Aedes aegypti surveillance and control: market assessment final deliverable Boston Consulting Group; 2017.
17. Wong S, Poon R, Wong S. Zika virus infection - the next wave after dengue? Journal of the Formosan Medical Association. 2016;115:226-42.
18. Wylie B, Hauptman M, Woolf A, Goldman R. Insect repellents during pregnancy in the era of the Zika virus. Obstetrics and gynecology. 2016;128.

Behavior 2: Use of condoms to prevent sexual transmission of Zika in pregnancy.

Summary of the Literature Findings:

- **Efficacy:**
  - The efficacy of condom use to prevent sexual transmission of ZIKV is assumed based on the ability of condoms to prevent transmission of STI's including HIV.
  - Studies have shown the sexual transmission of ZIKV (in macaques) to have high virulence meaning without condoms it is highly likely to transmit (Haddow et al, 2017). However, there is yet to be a study to assess the exact efficacy of condoms to stop the spread of ZIKV (Carvalho, 2016).
  - No studies of sexual transmission have been conducted in endemic countries (Carol Rao, communication).
  - All studies and guidelines reviewed from CDC[3], UNICEF[4], and WHO[5] all promote condom use as an effective prevention measure against sexual transmission of Zika, particularly during pregnancy. No studies to date have calculated the protective efficacy of condoms against ZIKV, however studies do show the virus is persistent in semen for at least 92 days, leading to the recommendation of 180 days of protected sex after a partner's infection (Duarte, 2017).
  - One study found women had a 90% higher chance of having ZIKV compared to men, suggesting male to female sexual transmission is the most likely cause for this gender disparity (however, the authors highlight this finding may reflect higher care-seeking by women or that women spend more time at home exposed to the vector); the authors recommend avoiding unprotected sexual intercourse (Coelho, 2017). A recent presentation also reported that sexual partners had increased risk of transmission, suggesting a role for sexual transmission in the epidemic (Rosenberg, 2017).
  - Mathematical modeling studies found the sexual transmission attack rate to range from 4-5% of total ZIKV transmission (Roa, 2017; Coelho, 2017). It is therefore thought to contribute little to the overall epidemic.
- **Potential to reduce Zika transmission at population level:**
  - Condom use will likely contribute only a small amount to reducing the overall transmission of ZIKV at the population level. However, recent findings suggest that for a reproductive-age, sexually-active woman, her attributable fraction for acquiring ZIKV by sex might be much higher (Rosenberg, 2017).
- **Frequency required to be effective:**
  - The frequency of application required to be effective was determined from the literature and field experience.
  - Condom use is highly effective when they are used correctly and consistently.
- **Feasibility of behavior:**
  - The feasibility of condom use was assessed from the literature and field experience.
  - Barriers to condom use found in the literature include: high levels of sexuality-related stigma, low levels of self-efficacy, poor quality sex education, low levels of reproductive planning, limited access to contraception, high levels of gender-based violence, low rates of

---

[3] CDC. Sexual transmission and prevention. Link: https://www.cdc.gov/zika/prevention/sexual-transmission-prevention.html

[4] UNICEF (2016). Risk Communication and Community Engagement for Zika Virus Prevention and Control. Link: https://www.unicef.org/cbsc/files/Zika_Virus_Prevention_and_Control_UNICEF_English.pdf

[5] World Health Organization (2016). Prevention of sexual transmission of Zika virus: Interim guidance update. Link: http://apps.who.int/iris/bitstream/10665/204421/1/WHO_ZIKV_MOC_16.1_eng.pdf

condom use among women, negative religious messaging around use of contraceptives, and limited participation of health centers to allocate these resources for prevention (Pacheco, 2017 ; Rodríguez-Díaz, 2017; Davis, 2016 ; Zorrilla, 2016; Hodge, 2016).
  - Studies reported low utilization of condoms generally but particularly during pregnancy (Marteleto, 2017; Zorrilla, 2017; D'Angelo et al 2017; Fraiz et al, 2018). One study in the US found 56% of pregnant women at risk of Zika rated condom use difficult (Fraiz, 2018). A recent survey in Puerto Rico found only 38.5% of pregnant women reported using condoms (D'Angelo, 2017).
- **Ease of access to materials required:**
  - The assessment of ease of access was based on field experience and the procurement of condoms by USAID.
  - No field studies identified regarding condom availability and pricing in USAID Zika response settings. One study in Brazil noted no change in the sales of contraceptive methods (including condoms and long acting reversible contraception) after Zika (Bahamondes, 2017).

List of Reviewed Literature:

1. Bahamondes L, Ali M, Monteiro I, Fernandes A. Contraceptive sales in the setting of the Zika virus epidemic. *Human Reproduction.* 2017;32(1):88-93.
2. Boggild AK, Geduld J, Libman M, et al. Surveillance report of Zika virus among Canadian travellers returning from the Americas. *Canadian Medical Association Journal.* 2017;189(9):E334-e340.
3. Citil Dogan A, Wayne S, Bauer S, et al. The Zika virus and pregnancy: evidence, management, and prevention. *J Matern Fetal Neonatal Med.* 2017;30(4):386-396.
4. Coelho FC, Durovni B, Saraceni V, et al. Higher incidence of Zika in adult women than adult men in Rio de Janeiro suggests a significant contribution of sexual transmission from men to women. *Int J Infect Dis.* 2016;51:128-132.
5. D'Angelo D, et al. Measures Taken to Prevent Zika Virus Infection During Pregnancy — Puerto Rico, 2016 | MMWR. *Morbidity and Mortality Weekly* 2017; https://www.cdc.gov/mmwr/volumes/66/wr/mm6622a2.htm.
6. De Carvalho NS, De Carvalho BF, Fugaça CA, Dóris B, Biscaia ES. Zika virus infection during pregnancy and microcephaly occurrence: a review of literature and Brazilian data. *The Brazilian Journal of Infectious Diseases.* 2016;20(3):282-289.
7. Doolabh K, Caviola L, Savulescu J, Selgelid M, Wilkinson DJ. Zika, contraception and the non-identity problem. *Developing World Bioethics.* 2017;17(3):173-204.
8. Duarte G, Moron AF, Timerman A, et al. Zika Virus Infection in Pregnant Women and Microcephaly. *Rev Bras Ginecol Obstet.* 2017;39(5):235-248.
9. Fellner C. Zika in America: The Year in Review. *P&T.* 2016;41(12):778-791.
10. Fraiz LD, de Roche A, Mauro C, et al. U.S. pregnant women's knowledge and attitudes about behavioral strategies and vaccines to prevent Zika acquisition. *Vaccine.* 2018;36(1):165-169.
11. Gao D, Lou Y, He D, et al. Prevention and Control of Zika as a Mosquito-Borne and Sexually Transmitted Disease: A Mathematical Modeling Analysis. *Sci Rep.* 2016;6:28070.
12. Haddaw A, et. al. High Infection Rates for Adult Macaques after Intravaginal or Intrarectal Inoculation with Zika Virus - Volume 23, Number 8—August 2017 - Emerging Infectious Disease journal - CDC. *Emerging Infectious Disease* 2017; https://wwwnc.cdc.gov/eid/article/23/8/17-0036_article.
13. Hodge JG, Corbett A, Repka A, Judd PJ. Zika Virus and Global Implications for Reproductive Health Reforms. *Disaster Med Public Health Prep.* 2016;10(5):713-715.

14. Maharajan MK, Ranjan A, Chu JF, et al. Zika Virus Infection: Current Concerns and Perspectives. *Clinical Review of Allergy & Immunology.* 2016;51(3):383-394.
15. Marteleto LJ, Weitzman A, Coutinho RZ, Valongueiro Alves S. Women's Reproductive Intentions and Behaviors during the Zika Epidemic in Brazil. *Population and Development Review.* 2018;43(2):199-227.
16. Maxian O, Neufeld A, Talis EJ, Childs LM, Blackwood JC. Zika virus dynamics: When does sexual transmission matter? *Epidemics.* 2017;21:48-55.
17. Moreira J, Peixoto TM, Siqueira AM, Lamas CC. Sexually acquired Zika virus: a systematic review. *Clin Microbiol Infect.* 2017;23(5):296-305.
18. Mukherjee, Reema Mukherjee[1] AK. Zika virus: Vaccine initiatives and obstacles. *Medical Journal of Dr DY Patil Vidyapeet.* 2017;10(1):10-15.
19. Nishiura H, Mizumoto K, Rock KS, Yasuda Y, Kinoshita R, Miyamatsu Y. A theoretical estimate of the risk of microcephaly during pregnancy with Zika virus infection. *Epidemics.* 2016;15:66-70.
20. Pacheco O, Beltrán M, Nelson CA, et al. Zika Virus Disease in Colombia — Preliminary Report. *The New England Journal of Medicine.* 2016.
21. Petersen LR, Jamieson DJ, Powers AM, Honein MA, Baden LR. Zika Virus. *New England Journal of Medicine.* 2016.
22. Rao R, Gaw SL, Han CS, Platt LD, Silverman NS. Zika Risk and Pregnancy in Clinical Practice: Ongoing Experience as the Outbreak Evolves. *Obstetrics and Gynecology.* 2017;129(6):1098-1103.
23. Rodriguez-Diaz CE, Garriga-Lopez A, Malave-Rivera SM, Vargas-Molina RL. Zika virus epidemic in Puerto Rico: Health justice too long delayed. *International Journal of Infectious Disease.* 2017;65:144-147.
24. Rosenberg ES, Doyle K, Munoz-Jordan J, Klein L, Adams L, Lozier M, et al., editors. Prevalence and incidence of Zika virus infection among household contacts of Zika patietns: Puerto Rico, 2016-2017. American Society for Tropical Medicine and Hygiene; 2017; Baltimore, MD.
25. Saiz JC, Vazquez-Calvo A, Blazquez AB, Merino-Ramos T, Escribano-Romero E, Martin-Acebes MA. Zika Virus: the Latest Newcomer. *Frontiers in Microbiology.* 2016;7:496.
26. Tambo E, Madjou G, Khayeka-Wandabwa C, Olalubi OA, Chengho CF, Khater EI. Ethical, legal and societal considerations on Zika virus epidemics complications in scaling-up prevention and control strategies. *Philos Ethics Humanit Med.* 2017;12.
27. Troncoso A. Zika threatens to become a huge worldwide pandemic. *Asian Pacific Journal of Tropical Biomedicine.* 2016;6(6):520-527.
28. Wahid B, Ali A, Rafique S, Idrees M. Zika: As an emergent epidemic. *Asian Pac J Trop Med.* 2016;9(8):723-729.
29. World Health Organization. WHO | Zika Strategic Response Plan. *WHO* 2016; http://www.who.int/emergencies/zika-virus/strategic-response-plan/en/.
30. World Health Organization WHO | Sexual transmission of Zika Virus: Current status, challenges and research priorities. 2017; http://www.who.int/reproductivehealth/zika/sexual-transmission-experts-meeting/en/.
31. Zorrilla CD, Mosquera AM, Rabionet S, Rivera-Vinas J. HIV and ZIKA in Pregnancy: Parallel Stories and New Challenges. *Obstetrics & Gynecology International Journal.* 2016;5(6).

Zika Prevention Behavior Matrix

Behavior 3: Regularly removing unintentional standing water both inside and outside of the house, and in communal areas.

Summary of the Literature Findings:

- **Efficacy:**
  - The efficacy of removing standing water is measured as reduced vector density of adult mosquitoes, since it removes the breeding site completely
  - In a quasi-experimental design study in Cuba, the house index was reduced from 3.7 to 0.61% (Perez, 2005).
  - In a meta-analysis, although findings pointed towards lower vector densities in households that cleared stagnant water, the main studies reviewed did not have sample size with sufficient power to detect differences as significant (Alvarado-Castro, 2017).
  - One study found that source reduction and education campaigns halved the presence of *A. albopictus*, which is also capable of carrying Zika, in intervention compared with control areas (Suter, 2016). However, another study did not find this effect unless they controlled for the number of containers, potentially because *A. albopictus* is able to breed in smaller, less obvious places if the larger containers or sources are removed (Dowling, 2013).
  - In Singapore, removal of stagnant water was associated with an over 70% reduction in adult mosquitoes, through a rigorous door to door campaign (Audraud, 2013).
  - Identification of, and targeted action towards, 'productive' container types (i.e. those that are assessed as contributing the greatest burden of pupae, relative to other containers in the area) can potentially enable more cost effective larval control (Bowman, 2016).
- **Potential to reduce Zika transmission at population level:**
  - The potential for reducing Zika transmission is based on the assumption that reducing breeding sites will reduce the population of adult mosquitoes, thus reducing disease transmission.
- **Frequency required to be effective:**
  - The frequency of application required to be effective was determined from the literature and field experience.
  - This intervention is most effective if the most productive containers are targeted, some studies aimed to identify what these are but may be context specific (Mahfodz, 2017).
  - A study found that even with source removal some households did not lower vector density because they inadvertently created other breeding sites in their yards by adding containers (Dowling, 2013).
- **Feasibility of behavior:**
  - The feasibility of this behavior was assessed from the literature and field experience.
  - Results from a qualitative study suggest the need for continued reinforcement of campaigns; despite reports indicating very high levels of knowledge, this study found that individuals would often forget or be too lazy to carry out the task (Wong, 2013). One study suggests that individuals are less likely to clear standing water if they think the government is carrying out spraying due to lowered perceived risk (Reyes Castro, 2017).
  - Another study found that even with source removal some households did not lower vector density because they inadvertently created other breeding sites in their yards by adding containers (Dowling, 2013).
- **Ease of access to materials required:**
  - The ease of access to materials was determined based on field experience. No specific materials are required to clear water sources at the household level.
    - In the community, additional resources may be necessary.

Zika Prevention Behavior Matrix

List of Reviewed Literature:

1. Adalja A. Lessons Learned during Dengue Outbreaks in the United States, 2001–2011 - Volume 18, Number 4—April 2012 - Emerging Infectious Disease journal - CDC. *Emerging Infectious Disease* 2012; https://wwwnc.cdc.gov/eid/article/18/4/11-0968_article.
2. Alvarado-Castro V, Paredes-Solís S, Nava-Aguilera E, et al. Assessing the effects of interventions for Aedes aegypti control: systematic review and meta-analysis of cluster randomised controlled trials. *BMC Public Health.* 2017;17(1).
3. Audraud M. A simple periodic-forced model for dengue fitted to incidence data in Singapore. - Abstract - Europe PMC. *Mathematical Biosciences.* 2013;244(1):22-24.
4. Bouzid M, Brainard J, Hooper L, Hunter PR. Public Health Interventions for Aedes Control in the Time of Zikavirus- A Meta-Review on Effectiveness of Vector Control Strategies. *PLoS Neglected Tropical Disease.* 2016;10(12):e0005176.
5. Bowman LR, Donegan S, McCall PJ. Is Dengue Vector Control Deficient in Effectiveness or Evidence?: Systematic Review and Meta-analysis. *PLoS Neglected Tropical Disease.* 2016;10(3):e0004551.
6. Bowman LR, Runge-Ranzinger S, McCall PJ. Assessing the relationship between vector indices and dengue transmission: a systematic review of the evidence. *PLoS Neglected Tropical Disease.* 2014;8(5):e2848.
7. Chandren JR, Wong LP, AbuBakar S. Practices of Dengue Fever Prevention and the Associated Factors among the Orang Asli in Peninsular Malaysia. *PLoS Negl Trop Dis.* 2015;9(8):e0003954.
8. Dowling Z, Armbruster P, LaDeau SL, DeCotiis M, Mottley J, Leisnham PT. Linking mosquito infestation to resident socioeconomic status, knowledge, and source reduction practices in suburban Washington, DC. *Ecohealth.* 2013;10(1):36-47.
9. Garcia-Betancourt T, Higuera-Mendieta DR, Gonzalez-Uribe C, Cortes S, Quintero J. Understanding Water Storage Practices of Urban Residents of an Endemic Dengue Area in Colombia: Perceptions, Rationale and Socio-Demographic Characteristics. *PLoS One.* 2015;10(6):e0129054.
10. Quintero J, Brochero H, Manrique-Saide P, et al. Ecological, biological and social dimensions of dengue vector breeding in five urban settings of Latin America: a multi-country study. *BMC Infectious Diseases.* 2014;14(1):38.
11. Le Goff G, Goodman SM, Elguero E, Robert V. Survey of the mosquitoes (Diptera: Culicidae) of Mayotte. *PLoS One.* 2014;9(7):e100696.
12. Lõhmus M, Balbus J. Making green infrastructure healthier infrastructure. In: *Infections and Ecological Epidemiology.* Vol 5.2015.
13. Ocampo CB, Mina NJ, Carabali M, Alexander N, Osorio L. Reduction in dengue cases observed during mass control of Aedes (Stegomyia) in street catch basins in an endemic urban area in Colombia. *Acta Tropica.* 2014;132:15-22.
14. Rajiah K, et al. Dengue in Asean countries: A simple review on prevalence and current approaches. *Indo American Journal of Pharmaceutical Research, 2014.* 2014;4(4).
15. Reyes-Castro PA, Castro-Luque L, Diaz-Caravantes R, Walker KR, Hayden MH, Ernst KC. Outdoor spatial spraying against dengue: A false sense of security among inhabitants of Hermosillo, Mexico. *PLoS Neglected Tropical Disease.* 2017;11(5):e0005611.
16. Roslan MA, Ngui R, Vythilingam I, Sulaiman WYW. Evaluation of sticky traps for adult Aedes mosquitoes in Malaysia: a potential monitoring and surveillance tool for the efficacy of control strategies. *Journal of Vector Ecology.* 2017;42(2):298-307.
17. Sanchez L, Perez D, Perez T, et al. Intersectoral coordination in Aedes aegypti control. A pilot project in Havana City, Cuba. *Tropical Medicine & International Health.* 2005;10(1):82-91.
18. Sarwar M. Source Reduction Practices for Mosquitoes (Diptera) Management to Prevent Dengue, Malaria and Other Arboborne Diseases - Semantic Scholar.

2015; https://www.semanticscholar.org/paper/Source-Reduction-Practices-for-Mosquitoes-(Diptera-Sarwar/f430749aceb4dacd56ef659caa67f48eb2bbd606.

19. Sen K, et al. Mosquito vector management knowledge, attitude, practices and future of user & environment friendly new generation botanical Mosquitocide formulations: A review (PDF Download Available). *International Journal of Chemical Studies.* 2017;5(3):32-37.
20. G. S, A. D. An awareness program on dengue fever among adults residing in an urban slum area, Coimbatore. *International Journal of Research and Medical Sciences.* 2017;5(12).
21. Suter TT, Flacio E, Feijoo Farina B, et al. Surveillance and Control of Aedes albopictus in the Swiss-Italian Border Region: Differences in Egg Densities between Intervention and Non-intervention Areas. *PLoS Neglected Tropical Disease.* 2016;10(1):e0004315.
22. Suwanbamrung C, Promsupa S, Doungsin T, Tongjan S. Risk factors related to dengue infections in primary school students: exploring students' basic knowledge of dengue and examining the larval indices in southern Thailand. *Journal of Infection and Public Health.* 2013;6(5):347-357.
23. Wong LP, AbuBakar S. Health beliefs and practices related to dengue fever: a focus group study. *PLoS Neglected Tropical Disease.* 2013;7(7):e2310.
24. Organization WH. WHO | Vector control operations framework for Zika virus. *WHO* 2016; http://www.who.int/csr/resources/publications/zika/vector-control/en/.
25. Mahfodz Z, Musa NN, Hasmi NA, Ismail HN, Pardi F. Potential breeding sites for Aedes albopictus and Aedes aegypti: assessment against different container types. *Journal of Fundamental and Applied Sciences.* 2017;9(6).
26. Zhang H, Georgescu P, Hassan AS. Mathematical insights and integrated strategies for the control of Aedes aegypti mosquito. *Applied Mathematics and Computation.* 2016;273:1059-1089.

Zika Prevention Behavior Matrix

Behavior 4: Covering water storage containers at all times with a cover that is tight fitting, and does not warp or touch the water.

Summary of the Literature Findings:

- **Efficacy:**
  - Efficacy of covering water storage containers using generic covers or insecticide treated covers were based on measured reductions of pupal or larval infestation in the containers.
  - A study in Mexico found covering water containers was associated with lower risk of presence of larvae or pupae (OR 0.22, 95% CI 0.15, 0.27) after controlling for Temephos use (Morales-Perez, 2017).
  - A second study from Thailand found correct covering associated with decreased larval infestation (Vannavong, 2017).
  - In an evaluation in Sri Lanka, water tank covers significantly reduced the number of tanks positive for immature stage *Ae. aegypti* (MD = -4.00; 95% CI -4.96, -3.04) (Bowman, 2016).
  - A study in Thailand found that correctly covering containers with lids was effective in reducing larval infestation (AOR: 0.1-0.25) when used on jars for storing drinking water (Phuanukoonnon, 2005). This study highlights that covering is not enough, but containers must be correctly covered: 34.9% larvae were identified in incorrectly or uncovered jars compared with 7.8% in containers covered correctly (Phuanukoonnon, 2005).
  - Some studies specifically explored insecticide treated covers, including insecticide treated nets. In one evaluation in Colombia, use of long-lasting insecticide treated net (LLTN) as lids reduced pupal infestation by 71% compared to 25% in control clusters (p<.01) - the larger the container the greater the effect (Quintero et al 2015). Similar effects were found in Cambodia also with LLTN's (Seng et al 2008). For LLTN magnitude of effect diminishes over time due to gradual reduction of insecticidal effect (Seng et al, 2008).
  - Potential interaction between frequency of container use and effectiveness of lids: in one study, they found that removing and replacing lids too often reduced effectiveness, suggesting they are more effective on containers used for longer term storage (Phuanukoonnon, 2005).
- **Potential to reduce Zika transmission at population level:**
  - The potential for reducing Zika transmission is an inferential assessment from the efficacy of reducing Aedes aegypti breeding sites. The studies identified for the most part did not link the intervention with Aedes aegypti borne diseases such as dengue, chikungunya, or Zika.
  - One cRCT in Vietnam explored dengue transmission after their intervention, including container covering, but found no effect (Tsunoda, 2013).
  - Generally, little evidence of quantifiable associations between vector indices and dengue transmission in the literature (Bowman, 2014).
- **Frequency required to be effective:**
  - The frequency of application required to be effective was determined from the literature and field experience.
  - In Thailand frequent use of a jar increased risk of larval infestation in the presence of a lid, but decreases it in jars without a lid (frequent = less than one week). (Phuanukoonnon, 2005).
- **Feasibility of behavior:**
  - The assessment of feasibility was based on field experience and the literature.
  - Container lids are not an absolute barrier and must be tightly fitted to prevent gravid females entering for oviposition (Vannavong, 2017).

- In one study of LLTN's as covers, use declined from 21.5% to 9.6% after 22 months, with the main reason being that the nets get dirty or damaged over time (Vanlerberghe et al 2011).

- **Ease of access to materials required:**
  - The assessment of ease of access was based on field experience.

List of Reviewed Literature:

1. Arunachalam N, Tyagi BK, Samuel M, Krishnamoorthi R, Manavalan R, Tewari SC, et al. Community-based control of Aedes aegypti by adoption of eco-health methods in Chennai City, India. Pathogens and global health. 2012;106(8):488-96.
2. Bowman LR, Donegan S, McCall PJ. Is Dengue Vector Control Deficient in Effectiveness or Evidence?: Systematic Review and Meta-analysis. PLoS Neglected Tropical Disease. 2016;10(3):e0004551.
3. Caprara A, Lima JW, Peixoto AC, Motta CM, Nobre JM, Sommerfeld J, et al. Entomological impact and social participation in dengue control: a cluster randomized trial in Fortaleza, Brazil. Transactions of the Royal Society of Tropical Medicine and Hygiene. 2015;109(2):99-105.
4. Kittayapong P, Thongyuan S, Olanratmanee P, Aumchareoun W, Koyadun S, Kittayapong R, et al. Application of eco-friendly tools and eco-bio-social strategies to control dengue vectors in urban and peri-urban settings in Thailand. Pathogens and global health. 2012;106(8):446-54.
5. Kroeger A, Lenhart A, Ochoa M, Villegas E, Levy M, Alexander N, et al. Effective control of dengue vectors with curtains and water container covers treated with insecticide in Mexico and Venezuela: cluster randomised trials. BMJ (Clinical research ed). 2006;332(7552):1247-52.
6. Maciel-de-Freitas R, Lourenco-de-Oliveira R. Does targeting key-containers effectively reduce Aedes aegypti population density? Tropical medicine & international health : TM & IH. 2011;16(8):965-73.
7. Morales-Pérez A, Nava-Aguilera E, Balanzar-Martínez A, Cortés-Guzmán AJ, Gasga-Salinas D, Rodríguez-Ramos IE, et al. Aedes aegypti breeding ecology in Guerrero: cross-sectional study of mosquito breeding sites from the baseline for the Camino Verde trial in Mexico. BMC Public Health. 2017;17(1).
8. Overgaard HJ, Alexander N, Matiz MI, Jaramillo JF, Olano VA, Vargas S, et al. A Cluster-Randomized Controlled Trial to Reduce Diarrheal Disease and Dengue Entomological Risk Factors in Rural Primary Schools in Colombia. PLoS Neglected Tropical Disease. 2016;10(11):e0005106.
9. Phuanukoonnon S, Mueller I, Bryan JH. Effectiveness of dengue control practices in household water containers in Northeast Thailand. Tropical medicine & international health : TM & IH. 2005;10(8):755-63.
10. Sanchez L, Perez D, Perez T, Sosa T, Cruz G, Kouri G, et al. Intersectoral coordination in Aedes aegypti control. A pilot project in Havana City, Cuba. Tropical medicine & international health : TM & IH. 2005;10(1):82-91.
11. Seng CM, Setha T, Nealon J, Chantha N, Socheat D, Nathan MB. The effect of long-lasting insecticidal water container covers on field populations of Aedes aegypti (L.) mosquitoes in Cambodia. Journal of vector ecology : journal of the Society for Vector Ecology. 2008;33(2):333-41.
12. Tsunoda T, Kawada H, Huynh TTT, Luu LL, Le SH, Tran HN, et al. Field trial on a novel control method for the dengue vector, Aedes aegypti by the systematic use of Olyset® Net and pyriproxyfen in Southern Vietnam. Parasites & vectors. 2013;6(1):6.
13. Vanlerberghe V, Villegas E, Jirarojwatana S, Santana N, Trongtorkit Y, Jirarojwatana R, et al. Determinants of uptake, short-term and continued use of insecticide-treated curtains and jar covers for dengue control. Tropical medicine & international health : TM & IH. 2011;16(2):162-73.
14. Vannavong N, Seidu R, Stenstrom TA, Dada N, Overgaard HJ. Effects of socio-demographic characteristics and household water management on Aedes aegypti production in suburban and rural villages in Laos and Thailand. Parasites & vectors. 2017;10(1):170.

Zika Prevention Behavior Matrix

Behavior 5: Eliminating mosquito eggs from walls of water storage containers weekly.

Summary of the Literature Findings:

- **Efficacy:**
  - Efficacy of cleaning water storage containers by scrubbing and rinsing the container was determined by changes in pupal or larval infestation in the containers.
  - A study in Colombia found monthly or never washed containers were 4 times more likely to be infested than those washed weekly (Overgaard, 2017).
  - In Thailand, infestation rates were 17.2% in containers cleaned weekly vs 39.1% in those cleaned monthly and 43.7% in those cleaned annually (Phuanukoonnon, 2005).
  - In addition to scrubbing, washing, rinsing the container, one RCT found an added effect using chlorine bleach to clean, called “La Untadita Method”. The RCT found that proper implementation of this cleaning method with chlorine bleach and 10 minutes of scrubbing with a brush (steps outlined in article) reduced infestation significantly compared to just manual cleaning (Fernandez, 1998).
- **Potential to reduce Zika transmission at population level:**
  - The potential for reducing Zika transmission is based on the assumption that reducing vector indices will reduce disease transmission.
  - Cleaning and draining jars of water once per week effectively killed larvae and pupae because time from larvae to adult takes 2 or more weeks; elimination of breeding in jars could reduce pupal production by approximately one-third, leading to reduced adult mosquito population and risk of disease transmission (Hiscox, 2013).
  - Generally, little evidence of quantifiable associations between vector indices and dengue transmission in the literature (Bowman, 2014).
- **Frequency required to be effective:**
  - The frequency of application required to be effective was determined from the literature and technical expertise from ZAP.
  - Weekly cleaning of water storage containers by emptying the container and scrubbing with a brush significantly reduced pupal infestation in several studies (Overgaard, 2017; Phuanukoonnon, 2005; Quintero, 2014; Hiscox, 2013).
- **Feasibility of behavior:**
  - The assessment of feasibility was based on field experience and the literature.
  - In the literature, some studies describe this behavior as less feasible because it requires emptying water storage containers frequently (weekly) in areas with insufficient or unreliable water access, that would make households less likely to carry this out (Wanti et al 2017; Garcia-Betancourt et al 2015; Suarez et al 2009)
- **Ease of access to materials required:**
  - The assessment of ease of access was based on field experience. All that is needed is a brush and potentially bleach or household cleaning products, which are common household items in the region.

List of Reviewed Literature:

1. Alobuia WM, Missikpode C, Aung M, Jolly PE. Knowledge, Attitude, and Practices Regarding Vector-borne Diseases in Western Jamaica. Annals of global health. 2015;81(5):654-63.
2. Arunachalam N, Tyagi BK, Samuel M, Krishnamoorthi R, Manavalan R, Tewari SC, et al. Community-based control of Aedes aegypti by adoption of eco-health methods in Chennai City, India. Pathogens and global health. 2012;106(8):488-96.

3. Bouzid M, Brainard J, Hooper L, Hunter PR. Public Health Interventions for Aedes Control in the Time of Zikavirus- A Meta-Review on Effectiveness of Vector Control Strategies. PLoS Neglected Tropical Disease. 2016;10(12):e0005176.
4. Fernandez EA, Leontsini E, Sherman C, Chan AS, Reyes CE, Lozano RC, et al. Trial of a community-based intervention to decrease infestation of Aedes aegypti mosquitoes in cement washbasins in El Progreso, Honduras. Acta tropica. 1998;70(2):171-83.
5. Garcia-Betancourt T, Higuera-Mendieta DR, Gonzalez-Uribe C, Cortes S, Quintero J. Understanding Water Storage Practices of Urban Residents of an Endemic Dengue Area in Colombia: Perceptions, Rationale and Socio-Demographic Characteristics. PloS one. 2015;10(6):e0129054.
6. Hiscox A, Kaye A, Vongphayloth K, Banks I, Piffer M, Khammanithong P, et al. Risk factors for the presence of Aedes aegypti and Aedes albopictus in domestic water-holding containers in areas impacted by the Nam Theun 2 hydroelectric project, Laos. The American journal of tropical medicine and hygiene. 2013;88(6):1070-8.
7. Overgaard HJ, Alexander N, Matiz MI, Jaramillo JF, Olano VA, Vargas S, et al. A Cluster-Randomized Controlled Trial to Reduce Diarrheal Disease and Dengue Entomological Risk Factors in Rural Primary Schools in Colombia. PLoS Neglected Tropical Disease. 2016;10(11):e0005106.
8. Overgaard HJ, Olano VA, Jaramillo JF, Matiz MI, Sarmiento D, Stenstrom TA, et al. A cross-sectional survey of Aedes aegypti immature abundance in urban and rural household containers in central Colombia. Parasites & vectors. 2017;10(1):356.
9. Paz-Soldan VA, Morrison AC, Cordova Lopez JJ, Lenhart A, Scott TW, Elder JP, et al. Dengue Knowledge and Preventive Practices in Iquitos, Peru. The American journal of tropical medicine and hygiene. 2015;93(6):1330-7.
10. Phuanukoonnon S, Mueller I, Bryan JH. Effectiveness of dengue control practices in household water containers in Northeast Thailand. Tropical medicine & international health : TM & IH. 2005;10(8):755-63.
11. Quintero J, Brochero H, Manrique-Saide, Barrera-Pérez M, Basso C, Romero S, et al. Ecological, biological and social dimensions of dengue vector breeding in five urban settings of Latin America: a multi-country study. BMC Infectious Diseases. 2014;14(1):38.
12. Suarez R, Gonzalez C, Carrasquilla G, Quintero J. An ecosystem perspective in the socio-cultural evaluation of dengue in two Colombian towns. Cadernos de saude publica. 2009;25:S104-14.
13. Suwanbamrung C, Promsupa S, Doungsin T, Tongjan S. Risk factors related to dengue infections in primary school students: exploring students' basic knowledge of dengue and examining the larval indices in southern Thailand. Journal of infection and public health. 2013;6(5):347-57.
14. Tran HP, Huynh TTT, Nguyen YT, Kutcher S, O'Rourke P, Marquart L, et al. Low Entomological Impact of New Water Supply Infrastructure in Southern Vietnam, with Reference to Dengue Vectors. The American journal of tropical medicine and hygiene. 2012;87(4):631-9.
15. Wanti W, Yudhastuti R, Yotopranoto S, Notobroto HB, Subekti S, Umniati SR. Container Positivity and Larva Distribution Based on the Container Characteristics. International Journal of Public Health Science. 2017;6(3):237-42.

Annex 2: Behaviors Not Selected for Further Review

It is worth noting some of the behaviors not selected for further review, for the reasons indicated below.

- **Sleeping under a mosquito net:** This behavior has limited efficacy, as most people sleep during the night and *Aedes aegypti* mosquitoes mainly bite during the day. This limits the time they may provide protection to during daytime naps, making their contribution to Zika prevention limited.[1] Additionally, USAID is not procuring mosquito nets for Zika.
- **Wearing long sleeves and pants:** In the climate where Zika is transmitted, implementing this behavior with sufficient consistency (all day, every day) is unlikely to be feasible, reducing its potential to make an important contribution to Zika prevention. There is also limited evidence that wearing regular clothing that has not been treated with insecticide is effective.
- **Wearing light colored clothing:** Implementing this behavior all day, every day is unlikely to be feasible and there is limited evidence that it is effective.
- **Applying larvicides:** While considered highly efficacious, larvicides should be applied by vector control technicians, rather than household members, so control over implementation of this behavior does not lie at the household level.
- **Applying larvivorous fish to water:** An intervention that is still in pilot phase; limited data available on efficacy. Additionally, USAID is not procuring larvivorous fish.
- **Using mosquito coils:** Efficacy appears limited upon initial review, with some studies even suggesting they increase dengue risk.[1]
- **Indoor residual spraying:** This behavior is implemented by vector control technicians and therefore does not lie under the control of the household. Limited literature on the efficacy.[1]
- **Planting basil:** While some research suggests that essential oils extracted from plants may have a repellent effect, no studies were identified that assess the repellent effect of having a basil plant.

---

[1] Bowman LR, Donegan S, McCall PJ (2016). Is Dengue Vector Control Deficient in Effectiveness or Evidence?: Systematic Review and Meta-analysis. PLoS Negl Trop Dis 10(3): e0004551. doi:10.1371/journal.pntd.0004551
