# Supplementary material for: Evidence-Based Process for Prioritizing Positive Behaviors for Promotion: Zika Prevention in Latin America and the Caribbean and Applicability to Future Health Emergency Responses
Source: Glob Health Sci Pract. 2019 Sep 23;7(3):404–17. doi: 10.9745/GHSP-D-19-00188 (PMC6816817; doi:10.9745/GHSP-D-19-00188)
Supplement: 19-00188-Pinchoff-Supplement2.pdf [file 19-00188-Pinchoff-Supplement2.pdf]

TECHNICAL SPECIFICATIONS CONTENT GUIDE
for Behaviors with High Potential to Prevent Zika

Updated: 8 August 2018

USAID FROM THE AMERICAN PEOPLE

Breakthrough ACTION + RESEARCH FOR SOCIAL & BEHAVIOR CHANGE

Acknowledgements

Breakthrough ACTION, with the United States Agency for International Development (USAID), would like to acknowledge UNICEF, PSI, Save the Children, IFRC, Abt Associates, MCDI, Global Communities, AMOS/SSI, CARE, the Pan American Development Fund, the Centers for Disease Control and Prevention, Population Council and the Johns Hopkins Center for Communication Programs for the technical expertise and feedback provided during the development of this content guide for Zika prevention.

This document is made possible by the generous support of the American people through the United States Agency for International Development (USAID). The contents are the responsibility of Breakthrough ACTION and do not necessarily reflect the views of USAID or the United States Government.

Contact: Priya.Parikh@jhu.edu

TECHNICAL SPECIFICATIONS CONTENT GUIDE
for Behaviors with the Greatest Potential to Prevent Zika

This guide accompanies the ***Zika Prevention Behavior Matrix***, which analyzes the efficacy and feasibility of 7 behaviors with high potential to prevent the transmission of Zika and Congenital Zika Syndrome, based on a review of existing literature, data, and consultation with implementing partners of the USAID Zika response in several countries of Latin America and the Caribbean. One of the findings of this analysis has been the need to be more specific in the promotion of prevention behaviors to maximize the potential effectiveness of each behavior. This level of specificity requires more detailed technical information and specific steps to facilitate the adoption of the desired behavior.

OBJECTIVE

The objective of the ***Technical Specifications Content Guide*** is to specify the evidence-based technical requirements and steps to be followed for the practice of each behavior to achieve the desired impact: reducing the risk of negative consequences caused by Zika during pregnancy.

HOW TO USE THIS GUIDE

As a first step to use this guide, it is recommended that users select behaviors from the ***Zika Prevention Behavior Matrix*** based on the priorities of each Zika response program and the specific country context.

This guide contains specific technical steps and requirements that should serve as an input for social and behavior change (SBC) strategies and for the construction of messages addressed to specific groups (for example, pregnant women). In addition, the guide includes recommendations for the promotion of each behavior based on recent experiences of Zika response programs. The user of this guide should adapt the technical content to their context, using local phrases and terms to ensure maximum understanding of how to carry out the prevention behaviors effectively. This technical content is an input for the development of activities and communication messages, which should be in turn developed to appeal to the strongest local motivators to achieve the desired behavior change. The more key actors in the response to Zika who use, adapt and incorporate the technical content of this guide, the greater the harmonization of technical information that is promoted, which is of vital importance to achieve mutual support and synergy of the various Zika prevention activities.

Behavior 1: *Application of **mosquito repellent** (DEET, Picaridin[1], IR3535, or oil of lemon eucalyptus[2], only), using each product as directed, for **duration of pregnancy**, to reduce risk of Zika transmission through mosquito bites.*

Repellent as a protective method against mosquito transmission of Zika

- Repellent use is one of the most effective and safe ways to protect a pregnant mother from the bites of the mosquito that transmits the Zika virus, decreasing the risk of infection to the baby.
- The use of repellent during pregnancy does not harm the baby.
- Repellent is different from insecticides that kill mosquitoes. Repellents are designed to keep mosquitoes away from people and prevent them from biting and only work if applied to the skin.
- The most effective, safe and recommended repellents by health authorities are those that contain one or more of the following active ingredients: DEET, Picaridin, IR3535 and oil of lemon eucalyptus.
- Homemade repellents do not have scientific evidence regarding their ability to sufficiently protect from mosquito bites.

Where to find repellents containing DEET, Picaridin, IR3535, or oil of lemon eucalyptus

- In some health centers, pharmacies or stores.
- Repellents can be found in the form of lotion, cream, gel or spray.

Repellent use

- In general, repellents come with instructions on how to use the product and the frequency with which to apply it. It is important to follow these directions.
- Repellent should be applied directly on skin that is not covered by clothing.
- Do not apply repellent directly to the face: repellent should be placed in one's hand and then applied to the face.
- Repellent should be applied several times a day, as indicated in the instructions printed on each type of repellent. Depending on the repellent, the following is recommended for application [fill in each box according to the products available in the given context]:

REPELLENT	Concentration	Recommended Frequency of Use
DEET	20%	Every ___ hours (specify)
DEET	30%	Every ___ hours (specify)
IR 3535	_______(specify)	Every ___ hours (specify)
Picaridin	_______(specify)	Every ___ hours (specify)
Oil of Lemon Eucalyptus	_______(specify)	Every ___ hours (specify)

- Repellent should be applied more frequently if the person sweats, takes a bath or changes clothes, ensuring that repellent is applied to the exposed skin.
- Avoid applying repellent to delicate and sensitive areas such as the eyes, mouth, nasal membranes, wounds, cuts, or irritated skin.
- If there is little repellent, the pregnant woman is the priority in the family for use of repellent.

Safe disposal of repellent

- Repellent bottles should never be reused or refilled
- Wrap empty repellent bottles to absorb any leaks, preferably using paper, and dispose of them in household garbage
- Never pour unused repellent down any drains (indoor or outdoor)
- Do not cut or burn empty repellent bottles

[1] Also known as Icaridin or Kbr3023

[2] Also known as para-methane-dios (PMD)

RECOMMENDATIONS FOR PROMOTION

- Priority groups for this behavior
    - Pregnant women
    - Partners of pregnant women
- Each repellent has its own specific instructions and at times these can be confusing. Therefore, when promoting repellent use, it is important to inform pregnant women that they can ask for guidance and counseling about the correct use of repellents from:
    - Health center staff when the pregnant mother attends prenatal care
    - Community health promotor
    - Pharmacist or manager of the pharmacy
- Any personnel who counsel pregnant couples about repellent use should share clear instructions about the correct and appropriate use of the repellent. They should also help them read the written instructions for the repellent in case they are confusing.
- The promotion of repellent use during pregnancy should **focus on the risk of Zika infection to the unborn baby as the main motivator**, since experiences indicate that the health of the baby and the risk of Zika Congenital Syndrome are the main factors that motivate the use of repellent by pregnant women, and for families to prioritize the pregnant woman for repellent use.

Behavior 2: *Use of* ***condoms during pregnancy*** *to prevent sexual transmission of Zika in pregnancy.*

Sexual Transmission of Zika

- Although Zika virus transmission occurs mainly through the bite of the *Aedes aegypti* mosquito, Zika can also be transmitted through unprotected sexual relations with a person infected with the Zika virus.
- Sexual transmission of Zika virus can occur through vaginal, anal or oral sex without protection.
- An infected person (male or female) can sexually transmit the Zika virus to their partner without protection even if they do not display symptoms, or if they have mild symptoms and these disappeared, and they may not even know they are infected with the virus.
- An infected mother can pass the Zika virus to her baby throughout the pregnancy. The infected baby can develop Zika Congenital Syndrome, which can include microcephaly.
- The Zika virus can stay in a man's semen for a long time, so condoms should be used for at least 3 months[3] after he was exposed to the mosquito that transmits the Zika virus if his partner is pregnant or planning a pregnancy.
- Partners who are sexually active during pregnancy can prevent sexual transmission of Zika virus through the correct use of condoms throughout the pregnancy each time they have vaginal, anal or oral sex.
- There is NO medicine or vaccine against the Zika virus.

Condoms prevent the transmission of Zika virus during sexual intercourse

- Pregnant couples should discuss with their partner about sexual transmission of the Zika virus and **negotiate the use of condoms during each sexual act throughout pregnancy** to protect from Zika virus.
- The other alternative to eliminate the risk of sexual transmission of the Zika virus during pregnancy is to abstain from sexual relations.

Correct use of condoms

What is a condom?

- A latex (elastic material) sleeve that is placed on the erect penis before each sexual relation.
- If sexually active during pregnancy, condom use is one of the best methods to prevent sexual transmission of the Zika virus.

How to use a condom correctly

- A new condom should be used in every sexual act and in each relationship.
- Before using the condom, make sure that the wrapper is not broken and that the expiration date is valid.
- A condom is placed when the penis is erect, before penetration, or in oral or sexual relations.
- The wrapper should be opened where the slit is, and using one's fingertips, not teeth or nails, which can damage the condom.
- When placing the condom, one should pinch the tip of the condom and then unroll it down to the base of the penis.

How to remove the condom

- After ejaculation, the condom should be removed while the penis is still erect.
- The condom should be removed by sliding it off the penis using toilet paper to avoid any contact with semen or vaginal fluid.
- The used condom should be wrapped in toilet paper and placed in the trash.
- If another sexual relation is desired immediately afterwards, a new condom should be used, following the same instructions to put it on and remove it from the penis.

How to protect the condom

- Do not carry condoms in a wallet or purse, as they can crush or break the wrapper and damage the condom.

---

[3] Update: Interim Guidance for Preconception Counseling and Prevention of Sexual Transmission of Zika Virus for Men with Possible Zika Virus Exposure — United States, August 2018

FIGURE 1: Steps for correct condom use

NOTE: Image will be included in the next version, of this guide, accompanied by the text below.

To prevent sexual transmission, use condoms correctly and consistently every time you have sex during your pregnancy.

1. Date

Check expiration date on the package, do not use if date has already passed.

2. Check

Make sure the wrapper is sealed and has not been damaged; to do this, press the wrapper to test for a small amount of air inside.

3. Open

Open wrapper with fingertips and remove condom; do not use scissors or your teeth as this could damage the condom.

4. Put it on

When putting the condom on the penis, make sure to leave an extra space empty of air at the tip of the condom for semen to collect.

5. Lubricate

Do not use petroleum jelly (Vaseline), cosmetic creams or oils as they can damage the condom; use only water-based lubricants.

6. Remove

After ejaculation, hold the ring of the condom at the base of the penis, removing it before losing the erection; then remove it completely taking care not to spill any semen and throw it in the garbage.

SOURCE: PASMO/PSI

RECOMMENDATIONS FOR PROMOTION

- Priority groups for this behavior:
  - Pregnant women
  - Partners of pregnant women
- It is important to **promote dialogue between pregnant couples**, motivating them to talk openly with each other about how the Zika virus can be transmitted through unprotected sex and the importance of preventing the sexual transmission of Zika virus during pregnancy.
- The health of the baby and the risk of Congenital Zika Syndrome to the baby should be reinforced as the main motivator for the couple to use condoms during pregnancy, since experience indicates that these are the main factors that motivate this behavior.

Behavior 3: *Regularly **removing standing water** not intended for storage around the home and in communal areas*

Eliminate standing water and breeding sites in the home

- *Aedes aegypti* is a very domestic mosquito, meaning it remains close to homes and to people, with flight distances between 200 and 500 meters. The female usually chooses several breeding sites in the same home or yard, wherever water has collected. As such, if there is a mosquito in the home, it is very likely that there are several breeding sites in the yard.
- The elimination of standing water (not intenged for storage), where mosquitoes lay their eggs, around the home and in the community, significantly reduces the adult mosquito population. If this activity is carried out at least once a week in the same areas, the adult mosquito population can be reduced by up to 70%.

Identification and elimination of *Aedes aegypti* breeding sites in the home

- There is much that can be done at the household level to reduce mosquitoes that threaten the family:
  - **Dedicate 15 minutes each week** to identify and eliminate the mosquito breeding sites around the the home and especially the yard. Look for any standing water (not intended for storage).
  - **Know that the most favorable places** for mosquito breeding are those containers exposed to rainwater, especially if under shade or where organic matter accumulates (i.e. leaves). These places are preferred by *Aedes aegypti* because the sun does not bother them and the organic matter in the water provide food for the larvae.
  - Be aware of the variation in the location of breeding sites when eliminating standing water from the yard, as mosquitoes tend to seek places of more difficult access to lay their eggs and thus resort to roofs and gutters.
- All objects that are exposed to rain or containers that may accumulate rainwater, around the home and its surroundings must be identified, including:

• Tires	• Tree trunks
• Gutters on roofs	• Pots, planters and their dishes/bases
• Empty plastic or glass bottles	• Toys
• Buckets/cubes	• Unusable objects
• Drinking dishes/troughs for animals	• Empty cans
• Bromeliads	• Vases
• Permanent or fixed objects such as: pilas, fountains, pools	• Others: [specify according to common objects in local context]

- Once these containers are identified, at least once a week they must be emptied and turned over, covered or thrown away. This is based on the life cycle of mosquitoes, whose larvae usually become adults between 7-10 days (see Figure 2).
- In the same way, the gutters on the roofs must be emptied and kept clean. If the flow of rainwater through gutters is very slow or obstructed, mosquitoes may also lay eggs in these structures.

Identification and elimination of Aedes aegypti breeding sites at **the community level**

- Removing standing water in the community should focus on where the breeding sites are located, **avoiding general community cleanings, as efforts are diluted** and less effective for eliminating mosquito breeding sites.
- **Mapping the breeding sites in the community** under the leadership of community authorities is a fundamental activity to ensure this behavior is focused on reducing the mosquito population.
    - The use of community maps is critical to focus the search on areas where stagnant water or rainwater tend to accumulate.
    - A **white plastic container** should be used to obtain and inspect water from each possible breeding site to see if it contains larvae.
    - It is recommended to focus the community search on schools, health posts, cemeteries, structures or houses under construction, empty lots and community centers.
    - Remember that the preferred places for *Aedes aegypti* are areas that accumulate rainwater, especially if shaded or where organic matter, such as dry leaves, accumulates. These places are preferred by *Aedes aegypti* because the sun does not bother them and the organic matter in the water provide food for the larvae.
- Once the areas with *Aedes* breeding sites have been identified, any container that may accumulate rainwater must be emptied and turned over, covered or thrown away.
- This elimination of mosquito breeding sites must be done weekly.
- Fumigation only kills adult mosquitoes that are flying at that time but does not attack eggs and larvae in breeding sites or adult mosquitoes that were not flying at the time of spraying.

FIGURE 2: Mosquito life cycle

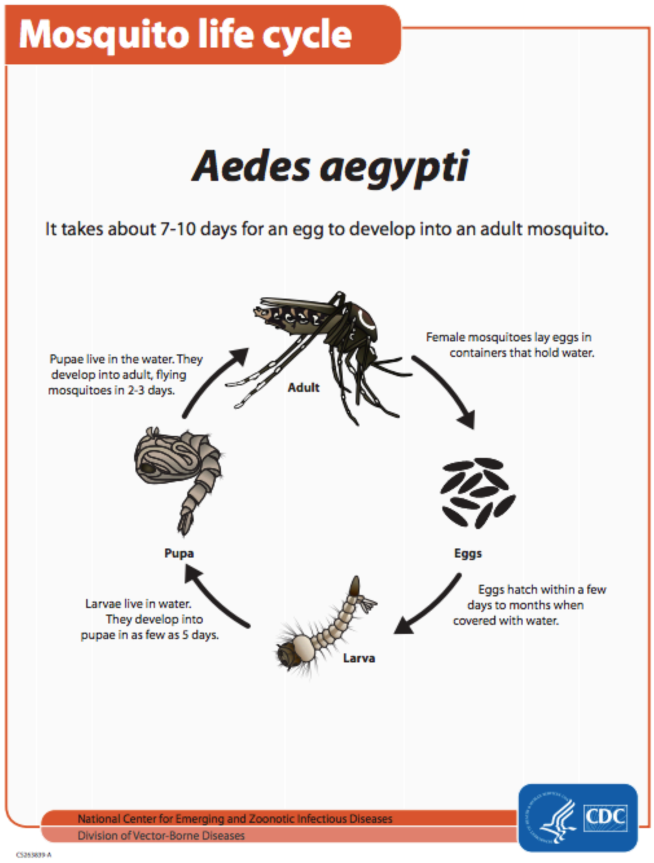

SOURCE: CDC

RECOMMENDATIONS FOR PROMOTION

Priority groups for this behavior

- Adults and children in the home
- Community leaders and neighborhood organizations

Identification and elimination of *Aedes aegypti* breeding sites <u>in the home</u>

- As a motivator to take action, explain that the breeding sites in the yard of the house are those that most put the family at risk.
- It is essential that technicians, health promoters, and community volunteers who conduct home visits **accompany family members through the house and yard** to identify breeding sites together, explain their life cycle and guide them on the technique to search, identify and eliminate *Aedes aegypti* larvae in water.
- To motivate action, it is advisable to empower the family with practical information about how to identify *Aedes aegypti* and the breeding sites that threaten their family:
    - Promote awareness of **the life cycle and behavior** of the *Aedes aegypti* mosquito for an active and more efficient search for eggs and larvae.
    - Promote an attitude of "**family detectives**" and encourage families to spend 15 minutes each week to identify and eliminate mosquito breeding sites in the yard of the house.
    - Inform families about the characteristics of **preferred breeding sites** of *Aedes aegypti* mentioned above.
    - Prromote being aware of any variation in the location of mosquito breeding sites around the home from week to week and pay attention to **roofs and gutters**.

Identification and elimination of *Aedes aegypti* breeding sites at <u>the community level</u>

- At the community level, this work is based on motivating and **mobilizaing action** by the community and its leaders to organize brigades to carry out the elimination of mosquito breeding sites.
- This effort requires a **pledge and weekly commitment** by community members/neighbors.
    - A **weekly breeding site search and elimination brigade** can be organized for this purpose.
    - **The efforts of neighbors can be recognized** at the community assembly for their work and positive results achieved.
- By encouraging community members to **share their learning** about the elimination of stagnant water with their neighbors, it increases the community commitment of everyone.

Behavior 4: *Covering infrequently used water storage containers* *at all times with a cover that is tight fitting and does not warp or touch the water*

Long-term water storage containers are potential breeding sites for *Aedes aegypti*

- Identify the **containers** in the home that are used to store **infrequently used water**. This is the water that is **accessed occasionally**, be it only once a week or even less frequently. Often, these are **fixed containers**. These may include:
  - Tanks, cisterns, barrels, water tanks, large buckets, and others
  - It is worth covering only the long-term fixed water storage containers and among them, those that present a more favorable environment for mosquitoes to lay their eggs (location in a shaded area, if organic matter falls into the water, such as leaves, etc.)
- Once the long-term water storage containers that could serve as breeding sites have been detected, evaluate if they can be covered with a tight-fitting lid

Type of lid to be used for fixed water storage containers

- Lids for long-term fixed water storage containers can block entrance of mosquitoes that seek to lay their eggs on the interior walls of these kinds of container.
- Not all lids are able to block mosquitoes from entering. For the lid to be effective, it's recommended that the lids have the following characteristics:
  - The lid should make a very tight seal. By not sealing well, the lid can have the opposite effect, providing shade over the water, making the water container more favorable to mosquitoes to lay eggs.
  - The lid should not touch the water, especially if it is a cloth cover because these can create small pools where the mosquito can lay its eggs.
  - If the cover is made of cloth, the fabric must allow rainwater to pass through it, so as to not accumulate water and convert the cover into a breeding site.
  - The lid must be made of a material that does not crack or warp in the heat or the sun.
  - A metal mesh with holes smaller than the size of an adult mosquito may be used, as long as it remains in perfect condition and seals tightly.
- A good lid is most effective when utilized **always, every day and every hour.**
- The lid must be kept in excellent condition and replaced if it begins to get warped or cracked.
- It is better not to cover the water containers, than to cover them partially or inconsistently.
- Ensuring a tightly sealed lid depends on the type of container. Some containers cannot be covered well, for example cement pilas, which are very difficult to cover completely. Barrels and round tanks are better suited for a lid that seals very tightly.
- The covering of long-term water storage containers is more effective when combined with scrubbing the internal walls of the container with detergent and with the elimination of mosquito breeding sites in communal areas.

RECOMMENDATIONS FOR PROMOTION

- Priority groups for this behavior
  - Members of the household in charge of long-term water storage containers
- The covers that have the greatest potential to be effective are those developed through a long-term intervention with community participation, in which covers are designed specifically for local containers that favor the development of mosquito breeding sites. It is important that the lids seal well and have all the features mentioned above.

Behavior 5: *Eliminating mosquito eggs* *from walls of* *frequently-used* *water storage containers weekly*

Frequently-used water storage containers are common breeding sites for *Aedes aegypti*

- In Central America, the most common containers that hold water for frequent use are "pilas" (cement washbasins) and water drums. In the Caribbean, the most frequently used water containers are plastic 45-65 gallon water drums.
- Large tanks or cisterns on roofs are usually tightly sealed and, therefore, do not apply to this behavior.
- Identify the containers in the home that are used to store water for frequent use. These are containers holding water that is used several times a week and thus the water tends to be stored for a relatively short time. These containers are frequently manipulated by users, both for accessing the stored water and to fill them up.
- Identifying these containers is important because the *Aedes aegypti* mosquito lays its eggs on the interior walls of these water containers and because these containers are often in close proximity to homes.
    - The female mosquito attaches eggs to the interior walls just above the water line.
    - The eggs are small and dark brown and can be difficult to see. A technician can guide individuals on how to identify eggs.
    - When the eggs hatch, the larvae develop and transform into pupae and then into adults, generally over a period of 7-10 days.
    - Sediment, leaves, algae and other types of organic material collect at the bottom of these containers and can serve as food for mosquito larvae.
- Once these frequently used water containers are identified, families should focus their efforts on these types of containers, as these containers are usually among the most important breeding sites in the household. It is necessary to set aside time every week to remove *Aedes aegytpi* eggs from the container walls.
- Simply "cleaning" the container is not enough to eliminate the eggs. The specific technique and materials utilized is crucial in order to reduce the number of eggs that may hatch.
- Scrubbing the walls of frequently used water storage containers is effective even in containers that have been treated with a larvicidal product.

Effective techniques for removing mosquito eggs from water container walls [ENTIRE SECTION IS NEW]

To develop these specifications, evidence was reviewed for four (4) techniques for eliminating *Aedes aegypti* eggs from the internal walls of water storage containers, such as "pilas" and water drums. Techniques 1–4 are recommended <u>in the order below</u>, based on the available evidence, potential for effectiveness, and feasibility of each one.

However, the feasibility of implementing these techniques varies by context. Please review the "FEASIBILITY AND CONTEXTUAL FACTORS TO CONSIDER" table below when deciding which technique, or group of techniques, to promote in a given context.

<u>Technique 1</u>: Scrubbing the walls of water storage containers with a brush and a mixture of non-ammonia detergent with bleach, following the Untadita method

Steps

1. Mix chlorine bleach with common powder laundry detergent (that does not contain ammonia) in equal parts to make a paste, do not add water.
2. Using a sponge, apply the mixture to the interior walls of the container, making up more mixture if necessary to cover the walls.
3. Wait at least 10 minutes for the bleach to have contact with the eggs.
4. Scrub the mixture on the walls with a hard plastic bristle bush for at least 5 minutes.

5. Rinse the walls of the container with water to remove the detergent and bleach mixture.

- The user may prefer to empty the container completely before applying the mixture (it does not have to be fully empty to begin)
- Repeat these steps **weekly**

Technique 2: Applying bleach directly to water storage container walls

Another technique to eliminate the eggs of *Aedes aegypti* is to apply household bleach to the walls of the container just above the water line.

Steps

1. Dip a sponge in household bleach. Undiluted bleach is recommended.
2. Dab the bleach on the interior walls of the container, focusing on the area above the water line. Ensure the area above the water line is well-covered with bleach.
3. If the container is empty of water, pour some bleach on the bottom as well.
4. Allow the bleach to dry for 15 minutes (allow for longer time if the bleach was diluted before applying).
5. Proceed with normal use of the water, or to fill the container as intended.

- **To be effective, it is important that the chlorine is placed directly onto the interior walls of the container and not poured into the water.**
- This technique only uses chlorine and no detergent or soap.
- This technique does not employ scrubbing the container walls.
- This technique does not require rinsing the container with water.
- This technique is not intended to purify drinking water nor to kill mosquito larvae living in the water. It is intended solely to kill eggs attached to the interior walls of the container when they are exposed to bleach as described above.

Technique 3: Scrubbing walls of the container with detergent

If the Untadita or bleach dabbing method cannot be feasibly practiced, scrubbing the internal container walls with detergent is recommended, following the steps below.

Steps

1. If possible, completely empty the water storage container and, also if possible, discard the water as it may contain larvae or pupae. If water is still in the container when it is scrubbed, eggs may become dislodged and sink to the bottom and may still hatch.
2. Using a brush with hard plastic bristles, with soap or detergent, scrub the internal walls of the container using a circular motion, especially the parts immediately above and below the line that was left by the water, to remove the eggs stuck to the walls.
3. If possible, scrub the bottom of the container to remove sediment, algae and leaves that accumulate and are food for mosquito larvae, making the container a less favorable environment for the mosquito.
4. The process should be repeated in a consistent manner, once a week, since the development of the mosquito from an egg to an adult generally takes between 7-10 days.

Technique 4: Scrubbing walls of the container with brush alone

Scrubbing alone is the least recommended technique but may be necessary if no detergent or bleach are available. The literature reports either no efficacy or mixed efficacy of this behavior on eliminating eggs but is better than not cleaning the container at all.

Steps

1. Completely empty the water storage container and, if possible, discard the water as it may contain larvae or pupae.

2. Once the container is empty, use a hard plastic bristle brush to remove the eggs from the interior walls by firmly scrubbing the walls of the container with circular motions.
3. Rinse the container thoroughly and throw out any remaining water once the container walls have been completely scrubbed.

- However, the use of the brush alone does not necessarily kill all the eggs. Eggs dislodged from the walls that fall into the water storage container may hatch if they were not destroyed or removed from the container by thorough rinsing.
- This technique should only be used when households are able to rinse out any remaining water once the container has been thoroughly scrubbed.

Localities with BTI (Bacillus thuringiensis) larvicide application

- In localities where the ZAP Project is applying larivicde (BTI), ZAP technicians visit each house monthly, alerting the community in advance of their arrival. The scrubbing of the walls of water containers containing BTI should be done shortly before the monthly visit to maximize the effect of the BTI between each ZAP technician visit.

FEASIBILITY AND CONTEXTUAL FACTORS TO CONSIDER

- The ranking of the four techniques listed above is based on available evidence and potential for efficacy.
- However, local context influences the feasility and apportipateness of each technique.
- Program planners may consult the table below in deciding which technique to promote for removal of *Aedes aegypti* eggs from walls of water storage containers.

**WATER SUPPLY**		**MATERIALS**	***Hard-bristle brush***	***Powder laundry detergent***	***Bleach***	**RECOMMENDED TECHNIQUE**
**Are households able to empty water storage containers on a weekly basis?**	Yes =>	**Are these materials generally available in homes?**	Yes	Yes	Yes	1 or 2
			Yes	Yes	No	3
			No	No	Yes	2
			Yes	No	No	4
		**MATERIALS**	***Hard-bristle brush***	***Powder laundry detergent***	***Bleach***	**RECOMMENDED TECHNIQUE**
Note: Container does not need to be empty to begin the cleaning process, but several techniques require rinsing completely. The emptied water may be reserved for rinsing the container.	No =>	**Are these materials generally available in homes?**	N/A	N/A	Yes	2

RECOMMENDATIONS FOR PROMOTION

- Priortiy groups for this behavior
  - Members of the home in charge of maintaining **frequently-used water storage containers**
- To encourage scrubbing of containers to become habitual, it is recommended that families choose one day each week that suits them best to carry out this process and repeat it consistently each week. Families may chose a day of the week that coincides with the arrival of the wáter supply to the home.
- Clarify with the people who are responsible for storing water in the family, how this technique of brushing to eliminate the eggs of *Aedes aegypti* is different from the general cleaning activities traditionally carried out in the home.

EVIDENCE REVIEW FINDINGS

Scrubbing only:

- WHO[4] recommends scrubbing alone but doesn't cite evidence or isolate the effect from other behaviors (such as using lids). They cite low uptake in many (Asian) settings.
- The Fernandez article (1998) says Untadita was developed because "manual cleaning was found to be ineffective in eliminating larvae". Work from the early 90s reported promotion of manual wash basin cleaning was not effective for failing to remove mosquito eggs (project staff reported people were cleaning algae not the eggs or missing eggs above the water line.)
- The Sherman et al (1998) article is more experimental/lab based but also found that brushing alone was not effective, partially due to eggs hatching from the brushes or at the bottom of the container if they are dislodged but not removed.
- **Finding: brushing alone – mixed or not effective; mixed feasibility.**

Untadita method:

- In one randomized trial by Fernandez et al (1998), Untadita was found to be more effective than scrubbing alone in its second iteration (after the messaging was simplified and the proportion of the bleach/detergent mixture modified to ½ bag chlorine bleach + ½ bag common laundry detergent).
- Field-based concerns regarding feasibility/safety of this behavior due to: potential toxicity should non-recommended types of detergent be mixed with chlorine, the need to thoroughly wash off detergent is difficult in water scarce settings, and also that it can't be applied to water containers that are not completely empty.
- **Finding: Untadita has high/mixed effectiveness; and high/mixed feasibility.**

Negociación de Prácticas Mejoradas (NEPRAM) trial of bleach application to water storage container walls:

- Leontsini et al (2004) implemented an innovative participatory methodology, i.e., developing the behaviors with a small group of community members to ensure feasibility. The final recommendation was to select one of two methods to perform once per week:
    1. Dab bleach directly on the entire walls of an emptied/washed container and to pour some into the bottom of the container (let sit for 15 mins);
    2. Dab bleach on the exposed walls of a container that is not fully empty.
- No field-based results on the effectiveness or scaling of these methods but the articles state the outcomes were "positive". An article by Albaine Pons (2002) mentions positive ovicidal findings in laboratory and semi-field tests, but is very small and experimental.
- **Finding: No data on effectiveness; high feasibility**

[4] http://www.wpro.who.int/mvp/documents/docs/Guidelines_for_dengue_surveillance_edition2.pdf; http://apps.searo.who.int/PDS_DOCS/B4957.pdf?ua=1

Behavior 6: *Seeking prenatal care* *to monitor pregnancy and discuss Zika risk and prevention*

The prenatal care visit: an opportunity to ask and learn for both the pregnant woman and her partner

- It is very important to attend prenatal checkups throughout pregnancy. They are a special opportunity to monitor pregnancy, clarify doubts and learn more about Zika and the risks for the pregnant mother and the baby that is on the way.
- During prenatal care, one can ask about an effective way to avoid sexual transmission of Zika by the correct use of condoms. Similarly, this is an opportunity to discuss the protection provided by the use of repellent to avoid mosquito bites.
- At prenatal care visits, pregnant women and their partners can feel confident in asking about how a pregnant woman infected with the Zika virus can pass the virus to her baby, who could in turn develop Zika Congenital Syndrome, which includes microcephaly.
- The prenatal care visit is also an opportunity for the partner to be informed, participate and accompany the pregnant mother.
- If a pregnant woman observes that her partner has any symptoms of Zika or if she thinks he may have been exposed to Zika, the couple should speak with a prenatal care service provider.

The prenatal care visit: an important opportunity for the health care provider

• The prenatal consultation is an opportunity to inform about the measures that a pregnant woman, her partner and her family can take to protect her baby from Congenital Zika Syndrome.

• The prenatal care provider can take advantage of the consultation time to promote the 5 behaviors with high potential to prevent Zika mentioned above, ensuring to provide very spefific steps and information to the pregnant woman and her partner on how to practice each behavior in the home.

RECOMMENDATIONS FOR PROMOTION

- Priority groups for this behavior
  - Pregnant women
  - Partners of pregnant women
  - Prenatal care providers
- It is important to promote dialogue between pregnant couples, encouraging them to speak openly with each other about Zika virus transmission to the baby during pregnancy.
- Attendance at prenatal appointments by the partner of the pregnant woman should be motivated, so they can both obtain invaluable information about the risk of Zika during pregnancy and, above all, how to prevent it.
- Reinforce the health of the baby and the risk of Congenital Zika Syndrome to the baby as the **main motivator** for the both the pregnant woman and her partner to attend the prenatal care visits and to adopt measures to prevent Zika during pregnancy.

Behavior 7: *Seeking counseling from a trained provider on* ***modern family planning methods*** *if not planning on getting pregnant*

Family planning as protection against the consequences of the Zika virus

- If one is sexually active and not planning to have a child, one can consult at a health facility about family planning options.
- For women or couples who wish to postpone pregnancy, access to family planning methods helps reduce the risks caused by Zika virus infection, and thus prevent Congenital Zika Syndrome affecting the baby, including microcephaly.
- The correct and consistent use of condoms confers double protection: it prevents an unplanned pregnancy and protects against the transmission of the Zika virus.
- There is NO medicine or vaccine against the Zika virus.

Access to family planning methods

- Family planning methods can be found at health service facilities, pharmacies and doctors' offices.

The most effective methods to prevent an unplanned pregnancy

- If pregnancy is not within one's plans, it is advisable to use a family planning method.
- Information and counseling can be obtained at the nearest health service, where one can discuss the most appropriate method for each person.
- There is a range of methods from which to choose. The most effective reversible methods to prevent an unplanned pregnancy and reduce the risk of mother-to-child transmission and therefore Congenital Zika Syndrome, are the hormonal implant, the IUD, injectables, pills and condoms.

Transmission of Zika through sexual intercourse

- Although transmission of Zika virus occurs mainly through the bite of the *Aedes aegypti* mosquito, it is also transmitted through the unprotected sexual intercourse, by an infected person (even without symptoms or very mild symptoms) to their partner.
- The Zika virus can remain in the semen for a long time therefore, condoms should be used for 6 months after being exposed to the mosquito that transmits Zika.
- An infected mother can pass the virus to her baby during pregnancy. The infected baby can develop Congenital Zika Syndrome which could lead to microcephaly.

RECOMMENDATIONS FOR PROMOTION

- Priority groups for this behavior
  - Women of reproductive age
  - Partners of women of reproductive age
- It is essential to promote dialogue between couples about family planning methods, motivating the couple to talk openly about family planning methods.
- The couple's conversation can also include dialogue on the correct and consistent use of the condom which confers double protection: it prevents an unplanned pregnancy and protects against the transmission of the Zika virus.
- Reinforce the health and well-being of the family, the realization of family aspirations, as well as the desire to reduce the risks of the Congenital Zika Syndrome, that could arise in the case of pregnancy, as the main motivators to practice this behavior.
